# CDCA7 is an evolutionarily conserved hemimethylated DNA sensor in eukaryotes

**DOI:** 10.1126/sciadv.adp5753

**Published:** 2024-08-23

**Authors:** Isabel E. Wassing, Atsuya Nishiyama, Reia Shikimachi, Qingyuan Jia, Amika Kikuchi, Moeri Hiruta, Keita Sugimura, Xin Hong, Yoshie Chiba, Junhui Peng, Christopher Jenness, Makoto Nakanishi, Li Zhao, Kyohei Arita, Hironori Funabiki

**Affiliations:** ^1^ Laboratory of Chromosome and Cell Biology, The Rockefeller University, New York, NY 10065, USA.; ^2^ Division of Cancer Cell Biology, The Institute of Medical Science, The University of Tokyo, Tokyo, Tokyo 108-8639, Japan.; ^3^ Structural Biology Laboratory, Graduate School of Medical Life Science, Yokohama City University, Yokohama, Kanagawa 230-0045, Japan.; ^4^ Laboratory of Evolutionary Genetics and Genomics, The Rockefeller University, New York, NY 10065, USA.

## Abstract

Mutations of the SNF2 family ATPase HELLS and its activator CDCA7 cause immunodeficiency, centromeric instability, and facial anomalies syndrome, characterized by DNA hypomethylation at heterochromatin. It remains unclear why CDCA7-HELLS is the sole nucleosome remodeling complex whose deficiency abrogates the maintenance of DNA methylation. We here identify the unique zinc-finger domain of CDCA7 as an evolutionarily conserved hemimethylation-sensing zinc finger (HMZF) domain. Cryo–electron microscopy structural analysis of the CDCA7-nucleosome complex reveals that the HMZF domain can recognize hemimethylated CpG in the outward-facing DNA major groove within the nucleosome core particle, whereas UHRF1, the critical activator of the maintenance methyltransferase DNMT1, cannot. CDCA7 recruits HELLS to hemimethylated chromatin and facilitates UHRF1-mediated H3 ubiquitylation associated with replication-uncoupled maintenance DNA methylation. We propose that the CDCA7-HELLS nucleosome remodeling complex assists the maintenance of DNA methylation on chromatin by sensing hemimethylated CpG that is otherwise inaccessible to UHRF1 and DNMT1.

## Teaser

CDCA7, activator of nucleosome remodeler HELLS, binds hemimethylated DNA to facilitate DNA methylation on chromatin.

## INTRODUCTION

DNA methylation is a broadly observed epigenetic modification in living systems, playing diverse functions in transcriptional regulation, transposable element silencing, as well as innate immunity (*1*). Alterations in DNA methylation patterns are linked to diseases such as cancers and immunodeficiency (*2*, *3*). One such disease is immunodeficiency, centromeric instability, and facial anomalies (ICF) syndrome. ICF patient cells exhibit hypomethylation of heterochromatic regions, such as satellite 2 repeats at the juxta-centromeric heterochromatin of chromosome 1 and 16 (*4*, *5*). Mutations in four genes are known to cause ICF syndrome: the de novo DNA methyltransferase *DNMT3B*, the SNF2-family adenosine triphosphatase (ATPase) *HELLS* (also known as *LSH*, *SMARCA6*, or *PASG*), the HELLS activator *CDCA7*, and the transcription factor *ZBTB24*, which is critical for the expression of CDCA7 (*6*–*8*). In addition, compound mutations of UHRF1, a critical regulator of maintenance DNA methylation, cause atypical ICF syndrome (*9*), supporting a further causal relationship between defective DNA methylation and the disease. The importance of HELLS and its plant ortholog DDM1 in DNA methylation has been established in vertebrates and in plants (*10*–*14*), and it has been suggested that the nucleosome remodeling activity of HELLS/DDM1 facilitates DNA methylation (*15*, *16*). However, it remains unclear why a role in promoting DNA methylation is uniquely carried out by HELLS/DDM1 among several other coexisting SNF2-family ATPases with similar nucleosome remodeling activity, such as SNF2 (SMARCA2/4), INO80, and ISWI (SMARCA1/5) (*17*).

In eukaryotes, DNA methylation is primarily observed as 5-methylcytosine (5mC), commonly in the context of CpG sequences, where both cytosines in the complementary DNA strands are symmetrically (i.e., fully) methylated. 5mC methylation mechanisms can be functionally classified as maintenance methylation or de novo methylation (*18*). Whereas de novo methylation, which is commonly mediated by DNMT3-family proteins, does not depend on preexisting 5mC on the template DNA, maintenance methylation, mediated by DNMT1-family proteins, occurs at hemimethylated CpGs, which are generated upon replication of fully methylated DNA. So far, the SET and RING-associated (SRA) domain of UHRF1 is the only established eukaryotic protein module that specifically recognizes hemimethylated CpGs (*19*–*21*). Through its E3 ubiquitin ligase activity, UHRF1 recruits and activates the maintenance DNA methyltransferase DNMT1 (*22*–*26*). During DNA replication, UHRF1-mediated dual mono-ubiquitylation of the proliferating cell nuclear antigen (PCNA)–associated factor PAF15 promotes DNMT1 activity to support DNA replication–coupled maintenance DNA methylation (*25*). In addition, when hemimethylated CpGs elude the imperfect replication-coupled maintenance methylation mechanism, DNMT1 can catalyze maintenance methylation far behind the replication fork. It has been suggested that this replication-uncoupled maintenance DNA methylation acts as a backup mechanism, which is most clearly observed in late-replicating/heterochromatin regions and is supported by UHRF1-mediated histone H3 dual mono-ubiquitylation, which activates DNMT1 (*7*, *25*, *27*, *28*). It was also shown that HELLS accelerates replication-uncoupled maintenance DNA methylation at late-replicating regions in HeLa cells (*27*). Furthermore, it has been reported that HELLS can assist the recruitment of UHRF1 and DNMT1 to chromatin and promote H3 ubiquitylation (*14*). While the observed HELLS-UHRF1 interaction may underlie the importance of HELLS in replication-uncoupled maintenance methylation (*14*), it remains unclear how HELLS is effectively recruited to sites of hemimethylation in this process.

The abundance of nucleosomes, which bend the DNA that wraps around the core histone octamer, affects the accessibility/activity of many DNA binding proteins (*29*), including DNA methyltransferases (*30*–*33*). The location of hemimethylated DNA within the nucleosome core particle (NCP) also inhibits its detection by the SRA domain of UHRF1 (*34*). In vivo, nucleosomal barriers to DNA methylation can be alleviated by the SNF2-family ATPase HELLS in vertebrates and DDM1 in plants (*15*). Although DDM1 can remodel the nucleosome on its own (*35*, *36*), we have previously demonstrated that HELLS alone is inactive and must bind CDCA7 to form the CDCA7-HELLS ICF–related nucleosome remodeling complex (CHIRRC), which exerts DNA-dependent ATPase and nucleosome remodeling activities (*16*). In *Xenopus* egg extracts, CDCA7 is critical for recruiting HELLS to chromatin, but not vice versa. CDCA7 also interacts with HELLS in human cells (*37*), and recruits HELLS to minor satellite DNA in mouse embryonic stem cells (*8*). The molecular basis of HELLS-CDCA7 interaction and CDCA7-chromatin interaction has not yet been established, however.

CDCA7 is characterized by its unique zinc-finger domain (pfam 10497; zf-4CXXC_R1), which is broadly conserved in eukaryotes (fig. S1) (*17*). CDCA7 homologs with the prototypical zf-4CXXC_R1 domain, containing 11 highly conserved signature cysteine residues and three ICF disease–associated residues, are almost exclusively identified in species that also harbor HELLS/DDM1 and maintenance DNA methyltransferases (DNMT1/MET1 or DNMT5), whereas CDCA7 is almost always lost in species that lack detectable genomic 5mC, such as *Drosophila*, *Tribolium*, *Microplitis*, *Caenorhabditis*, *Schizosaccharomyces pombe*, and *Saccharomyces cerevisiae* (*17*). This coevolution analysis suggests that the zf-4CXXC_R1 domain became readily dispensable in species that lack methylated DNA (*17*). However, the function of zf-4CXXC_R1 remains to be defined. We demonstrate that the zf-4CXXC_R1 domain of CDCA7 is a sensor for hemimethylated DNA, here referred to as the hemimethylation-sensing zinc finger (HMZF) domain. Unlike the SRA domain of UHRF1, the HMZF domain can recognize hemimethylated CpGs positioned within the NCP. Our results help explain how CDCA7 could confer the unique role of HELLS in maintenance DNA methylation by sensing hemimethylated CpG within the NCP.

## RESULTS

### CDCA7 selectively binds hemimethylated DNA

**Fig. 1.**
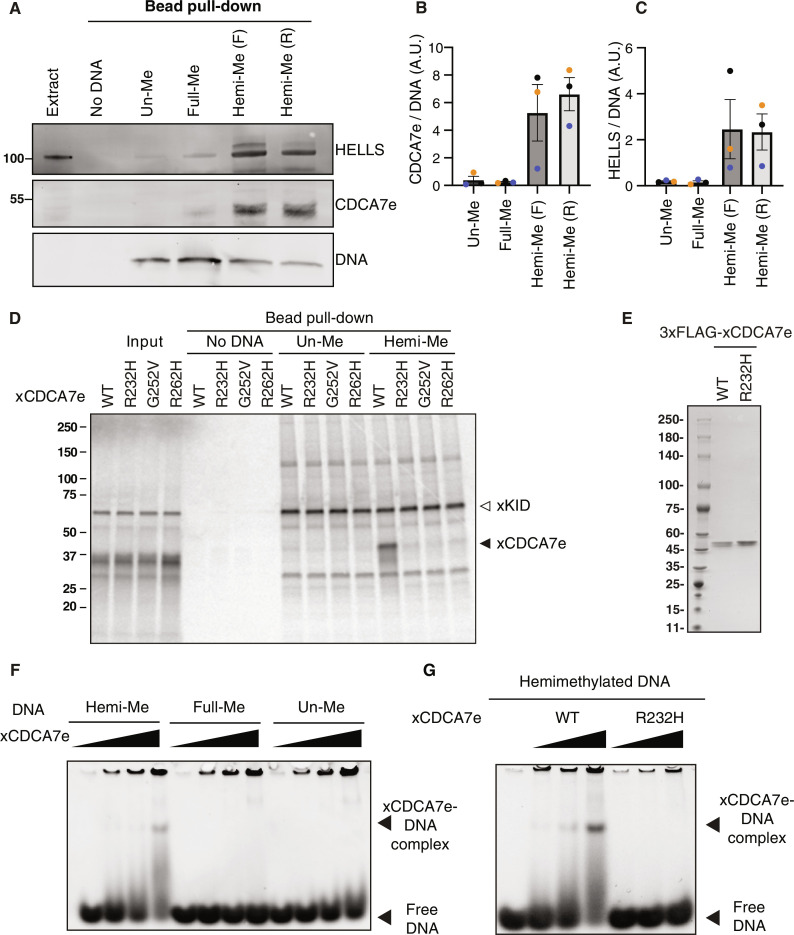
CDCA7 selectively binds hemimethylated DNA. (**A**) Magnetic beads coupled with double-stranded 54-bp DNA oligos containing unmethylated CpGs (un-Me), fully methylated CpGs (full-Me), or hemimethylated CpGs [hemi-Me; (F) and (R) to indicated 5mC in the forward- or reverse- strand; table S1], were incubated with interphase *Xenopus* egg extracts. Beads were collected after 10 min and analyzed by Western blotting. SDS–polyacrylamide gel electrophoresis (SDS-PAGE) was stained with SYBR Safe to visualize loading of the 54-bp DNA. Representative of *n* = 3 independent experiments. (**B**) Quantification of CDCA7e signal in Western blot analyses described in (A). CDCA7e signal at the DNA beads is normalized relative to the DNA signal. A.U., arbitrary units. *n* = 3 (biological replicates). The means and SEM are shown. (**C**) Quantification of HELLS signal in the Western blot analyses described in (A). HELLS signal at the DNA beads is normalized relative to the DNA signal. *n* = 3 (biological replicates). The means and SEM are shown. (**D**) ^35^S-labeled *X. laevis* CDCA7e proteins (wild type or with the indicated ICF3-patient associated mutation) were incubated with control beads, or beads conjugated 200-bp unmethylated or hemimethylated DNA (table S1). ^35^S-labeled xKid (*80*), a nonspecific DNA binding protein, was used as a loading control. Autoradiography of ^35^S-labeled proteins in input and beads fraction is shown. (**E**) Coomassie staining of purified 3xFLAG-tagged CDCA7e^WT^ and CDCA7e^R232H^ used in (F) and (G). (**F** and **G**) EMSA using recombinant *X. laevis* (F) CDCA7e^WT^ and (G) CDCA7e^R232H^. In graphs, data points from each biological replicate are annotated in a unique color.

CDCA7-family proteins are defined by the presence of a unique zinc-finger domain (zf-4CXXC_R1), in which all three identified ICF disease–associated residues are highly conserved (fig. S1) (*17*). CDCA7 homologs have coevolved with HELLS and the maintenance DNA methyltransferases, but not DNMT3-like de novo methyltransferases, suggesting a mechanistic link between CDCA7, HELLS, and maintenance DNA methylation at hemimethylated DNA (*17*). Since CDCA7e (the sole CDCA7 paralog present in *Xenopus* eggs) recruits HELLS to chromatin in *Xenopus* egg extracts but not vice versa (*16*), we explored the possibility that CDCA7e directly recognizes hemimethylated DNA. To test this hypothesis, beads coupled with unmethylated, hemimethylated, or fully methylated CpG–containing double-stranded 54–base pair (bp) DNA were incubated with *Xenopus* egg extracts. Notably, both CDCA7e and HELLS were markedly enriched on hemimethylated DNA over unmethylated or fully methylated DNA (Fig. 1, A to C, and table S1). When ^35^S-labeled *Xenopus laevis* CDCA7e produced in reticulocyte lysates was assessed for its DNA binding in vitro, wild-type CDCA7e but not CDCA7e with any of the ICF disease–associated mutations (R232H, G252V, or R262H) selectively associated with hemimethylated DNA (Fig. 1D and table S1). Direct and specific binding of CDCA7e to hemimethylated DNA was further confirmed by electrophoretic mobility shift assay (EMSA) using purified recombinant protein and double-stranded oligo-DNA containing a single hemimethylated CpG site (Fig. 1, E to G).

### HELLS and CDCA7 enrichment on hemimethylated chromatin

We next examined whether CDCA7e and HELLS are enriched on hemimethylated DNA in the context of chromatin. CDCA7e binds hemimethylated 3-kb DNA beads chromatinized in egg extract with similar specificity as UHRF1 (fig. S2A). As expected, higher molecular weight H3 species, characteristic for mono- and di-ubiquitylation of H3 by UHRF1, can be distinguished on the hemimethylated substrate.

Enrichment of CDCA7e and HELLS on hemimethylated DNA was also seen on native chromatin substrates. Adding sperm nuclei to egg extracts promotes functional nuclear formation, upon which DNA replication is rapidly executed between 30 and 60 min after incubation (*38*). Replication of the highly methylated sperm chromatin transiently generates hemimethylated DNA, which induces maintenance DNA methylation by UHRF1 and DNMT1 (*24*–*26*). When maintenance methylation is inhibited, hemimethylated DNA is expected to accumulate during DNA replication. To inhibit maintenance methylation, we used recombinant mouse DPPA3 (mDPPA3), which binds to UHRF1 and inhibits its association with chromatin (*39*, *40*). In control egg extracts, DNMT1, UHRF1, HELLS, and CDCA7e transiently associated with chromatin in S phase (40 to 60 min after sperm nucleus addition to egg extracts) (fig. S2B). In the presence of mDPPA3, DNMT1 and UHRF1 failed to associate with chromatin, while CDCA7e and HELLS exhibit robust and continuous chromatin accumulation during the time course (fig. S2B). These results support the idea that CDCA7e and HELLS are enriched on highly hemimethylated chromatin generated upon DNA replication in the absence of active maintenance DNA methylation. Consistent with this idea, chromatin association of CDCA7e and HELLS was suppressed when DNA replication was inhibited by geminin (fig. S2C) (*41*). Since CDCA7 and HELLS recruitment is observed even when mDPPA3 depleted UHRF1 from chromatin, this rules out the possibility that CDCA7 and HELLS recruitment to hemimethylated DNA is mediated by UHRF1. Of note, although it has been reported that UHRF1 and HELLS interact (*14*), we failed to detect measurable interaction between UHRF1 and HELLS in *Xenopus* egg extracts by reciprocal coimmunoprecipitation assays (fig. S2, D and E).

### Selective recognition of hemimethylated DNA by the CDCA7 HMZF domain is evolutionarily conserved

**Fig. 2.**
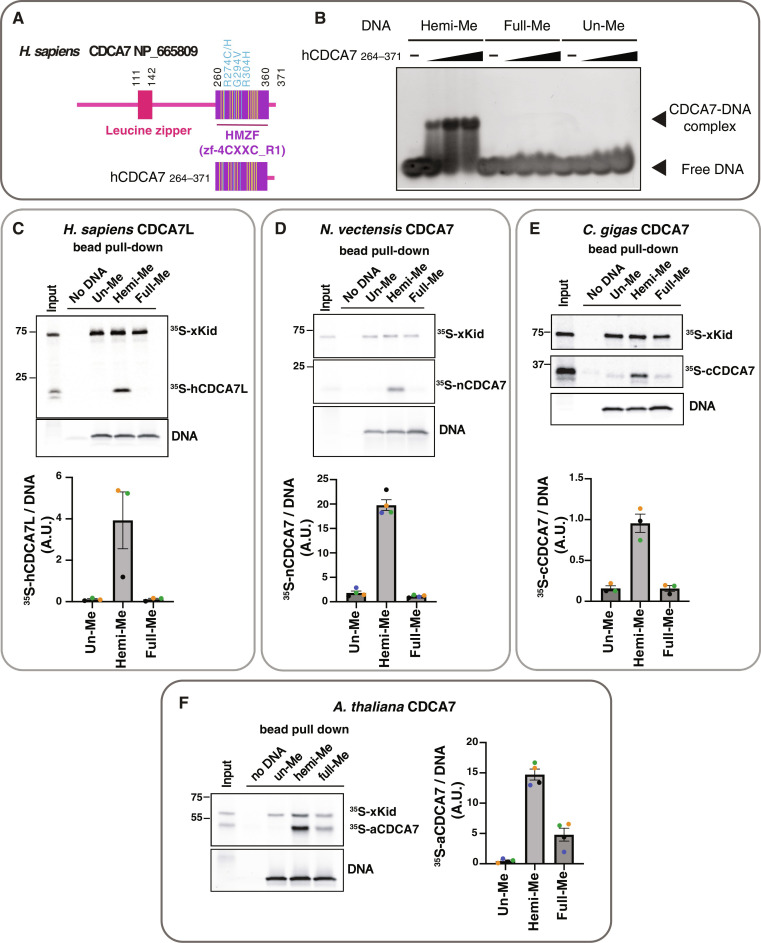
Selective binding of hemimethylated CpG by the HMZF domain of CDCA7 is evolutionarily conserved. (**A**) Schematic of *H. sapiens* CDCA7 (isoform 2 NP_665809). Positions of the HMZF (zf-4CXXC_R1) domain (purple), three ICF3-patient mutations (cyan), and conserved cysteine residues (yellow) are shown. (**B**) EMSA assay using the purified HMZF domain (amino acids 264 to 371) of *H. sapiens* CDCA7. (**C** to **F**) Magnetic beads coupled with double-stranded 54-bp DNA oligos containing unmethylated (un-Me), hemimethylated (hemi-me), or fully methylated (full-Me) CpGs were incubated for 10 min in the presence of the indicated ^35^S-labeled CDCA7 homolog and ^35^S-labeled xKid proteins. SDS-PAGE gels were stained with SYBR-Safe to visualize loading of the 54-bp DNA. Representative autoradiographs of ^35^S-labeled proteins in input and DNA pull-downs are shown. Quantifications of pulled-down ^35^S CDCA7 signal relative to the DNA signal are shown in a bar graph indicating the mean with SEM. In graphs, data points from each biological replicate are annotated in a unique color. The means and SEM are shown. (C) Pull-down of ^35^S-labeled human CDCA7 paralog CDCA7L (amino acids 322 to 454 of NP_061189) from *Xenopus* egg extract. Bar graph shows the quantification of data from *n* = 3 independent experiments. (D) Pull-down of ^35^S-labeled *N. vectensis* CDCA7 homolog (EDO33918.1) from boiled and clarified *Xenopus* egg extract supernatant. Bar graph shows the quantification of data from *n* = 4 independent experiments. (E) Pull-down of ^35^S-labeled *C. gigas* CDCA7 homolog (XP_011438013) from boiled and clarified *Xenopus* egg extract supernatant. Bar graph shows quantification of data from *n* = 3 independent experiments. (F) Pull-down of ^35^S-labeled *A. thaliana* CDCA7 homolog (NP_195428) from boiled and clarified *Xenopus* egg extract supernatant. Bar graph shows quantification of data from *n* = 4 independent experiments.

Hemimethylated DNA–specific binding was also observed for human CDCA7. Using the recombinant zf-4CXXC_R1 domain of human CDCA7 (Fig. 2A and fig. S3, A and E), we found that the cysteine-rich segment (amino acids 264 to 340 of NP_665809) of the domain alone does not exhibit any detectable DNA binding capacity (fig. S3B). Adding an N-terminal extension (amino acids 235 to 263) to the cysteine-rich segment weakly increased binding to the oligo-DNA with a hemimethylated CpG (fig. S3C). However, extending the cysteine-rich segment to include the evolutionarily conserved C terminus (amino acids 341 to 371), which contains two predicted alpha helices, conferred highly selective hemimethylation-dependent DNA binding (Fig. 2B). Therefore, we refer to the zf-4CXXC_R1 domain (corresponding to highly conserved amino acids 260 to 360 of NP_665809; figs. S1 and S3A) as the HMZF domain.

To assess evolutionary conservation of the observed hemimethylated DNA selectivity, CDCA7 homologs from various species were further tested for their DNA binding preference. Similar to human CDCA7, the HMZF domain of human CDCA7 paralog CDCA7L (amino acids 322 to 454 of NP_061189), displayed preferential binding to hemimethylated DNA (fig. S1 and Fig. 2C). Hemimethylated DNA–selective binding was also observed with invertebrate CDCA7 homologs of the sea anemone *Nematostella vectensis* (EDO33918.1) and the pacific oyster *Crassostrea gigas* (XP_011438013), as well as a homolog of the plant *Arabidopsis thaliana* (NP_195428) (Fig. 2, D to F). While all CDCA7 homologs tested displayed notably increased binding to hemimethylated DNA over both unmethylated or fully methylated DNA, a weak but reproducible preference for fully methylated DNA over unmethylated DNA was detected for *A. thaliana* CDCA7 in this DNA pull-down assay (Fig. 2F). Together, these results demonstrate that the HMZF domain of CDCA7 acts as a highly selective hemimethylated DNA binding module and suggest that this characteristic is evolutionarily conserved in plants and animals.

### CDCA7 recognizes a hemimethylated CpG at the major groove of linker DNA

**Fig. 3.**
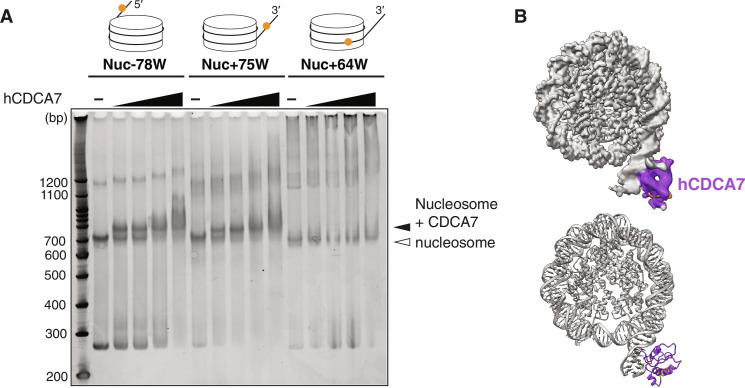
Cryo-EM structure of hCDCA7 bound at linker DNA. (**A**) EMSA analyzing the interaction of hCDCA7_264–371_ C339S with nucleosomes carrying hemimethylated CpG at the indicated positions. (**B**) A composite cryo-EM map (top) and the model structure (bottom) of hCDCA7_264–371_ C339S (generated from AF2) bound to Nuc+75W shown in (A). The map corresponding to CDCA7 is colored purple except for the conserved C-terminal helix, which is colored orange.

Since CDCA7 stimulates nucleosome remodeling activity of HELLS, we asked how the nucleosome could affect recognition of hemimethylated CpG by CDCA7. To address this question by biochemical and structural approaches, we generated the recombinant HMZF domain of human CDCA7 (hCDCA7_264–371_ C339S). The C339S substitution was included to improve protein homogeneity during purification while maintaining robust hemimethylated CpG–specific binding (fig. S3, D and E); C339 is not broadly conserved in CDCA7 family proteins and is substituted to serine in *X. laevis* CDCA7e and *C. gigas* CDCA7 (fig. S1) (*17*). EMSA demonstrated that the nucleosome-hCDCA7_264–371_ complex was readily observed when a hemimethylated CpG was positioned at the linker DNA at either the 5′ or 3′ end [Nuc-78W or Nuc+75W, annotated by the base position at the Watson (W) or Crick (C) strand, where position 0 indicates the dyad] (Fig. 3A and table S2). However, the complex formation was undetectable when the hemimethylated CpG was located within the NCP (Nuc+64W; Fig. 3A and table S2).

To gain structural insight into CDCA7-hemimethylated DNA interaction, cryo–electron microscopy (cryo-EM) single-particle analysis was conducted on hCDCA7_264–371_ C339S in complex with Nuc+75W (figs. S4 and S5 and tables S2 table S3). The initial cryo-EM map showed a density around the major groove of the hemimethylated CpG in the linker DNA (fig. S4). Three-dimensional (3D) variability analysis and 3D classification generated a cryo-EM map of 3.18-Å resolution for the NCP, where core histones and the phosphate backbone of DNA were clearly resolved. Local refinement and local classification generated a 4.83-Å resolution map for the extra cryo-EM density located outside of the linker DNA, representing hCDCA7_264–371_ C339S (Fig. 3B and figs. S4 and S5). A provisional structural model of linker DNA-bound hCDCA7_264–371_ was generated by fitting an AlphaFold2 (AF2)–predicted structure to the extra cryo-EM density at the linker DNA (fig. S5C) (*42*, *43*). However, the extra density remained somewhat ambiguous as the AF2-predicted structure of the HMZF domain could not fully account for the observed cryo-EM density.

### CDCA7 can recognize a hemimethylated CpG at the major groove of the NCP

Despite its low resolution, the cryo-EM map of the linker DNA-bound hCDCA7_264–371_ suggested that the HMZF domain primarily contacts hemimethylated CpG in the major groove of the DNA (Fig. 3B). This contrasts with the recognition of hemimethylated CpG by the SRA domain of UHRF1, which involves extensive engagement of the DNA at both the major and minor grooves and base-flipping of 5mC (*19*–*21*). Given the abundant histone-DNA contacts within the minor groove of the NCP, SRA binding to hemimethylated CpGs is necessarily obstructed within the NCP (fig. S6A) (*34*). We hypothesized that the binding mode of CDCA7 may therefore be more amenable to detecting hemimethylated CpGs within the NCP.

**Fig. 4.**
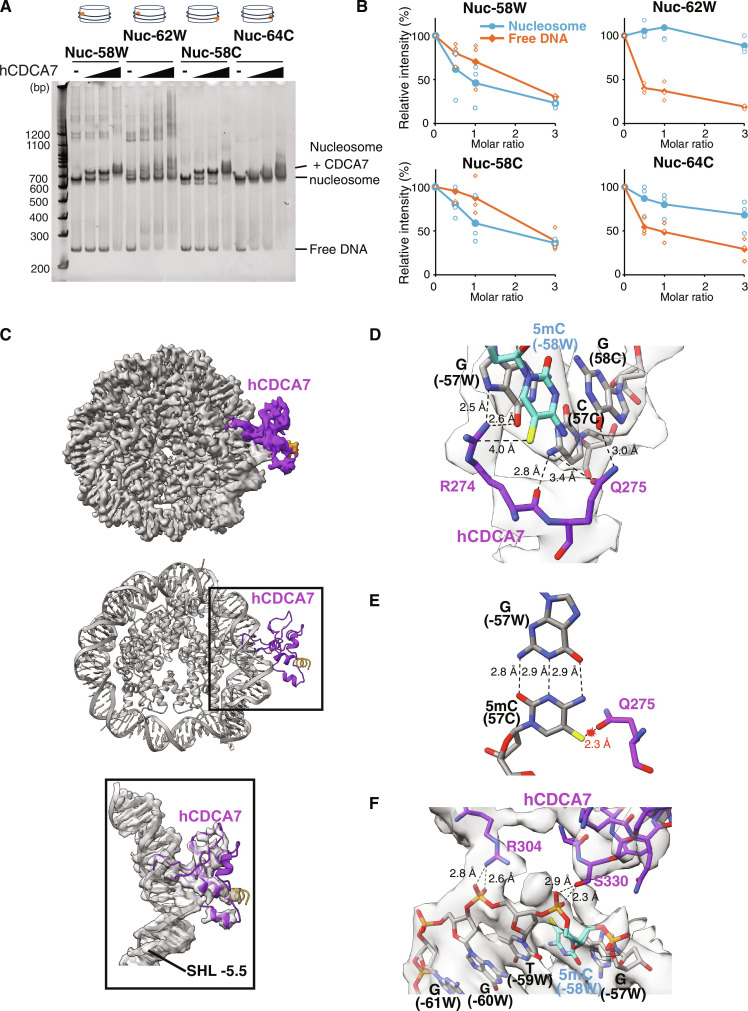
Cryo-EM structure of hCDCA7 bound at NCP. (**A**) EMSA analyzing the interaction of hCDCA7_264–371_ C339S with nucleosomes carrying hemimethylated CpG at the indicated positions. (**B**) Quantification of the free DNA (orange) and nucleosome signal (blue) detected by EMSA upon the addition of hCDCA7_264–371_ C339S relative to the signal detected in the absence of CDCA7. Line graph shows the average from *n* = 3 independent experiments. (**C**) A composite cryo-EM map (top) and the structure of hCDCA7_264–371_ C339S (middle) bound to the nucleosome harboring a 5mC at the Watson strand, position -58 (Nuc -58W). The map corresponding to CDCA7 is colored purple, conserved C-terminal helix indicated in orange. Overlay of atomic model of hCDCA7_264–371_ C339S on the cryo-EM map (bottom). (**D**) A structure of hCDCA7_264–371_ C339S bound to the nucleosome (Nuc -58W). Key residues for the selective recognition of hemimethylation, R274 and Q275, are shown as purple stick model. The methyl group of 5mC is shown in yellow. (**E**) Predicted steric clash between Q275 and the methyl group at the Crick strand, 5mC position 57 in a nucleosome harboring a fully methylated CpG dyad (5mC -58 W/5mC 57C) (**F**) A structure of hCDCA7_264–371_ C339S bound to the nucleosome (Nuc -58 W). Residues contacting the phosphate backbone of DNA are shown.

To explore this hypothesis, the provisional structural model of linker DNA-bound hCDCA7_264–371_ was superimposed at different positions along the NCP to predict where CDCA7 may recognize a hemimethylated CpG (figs. S5C and S6, B and C). Consistent with the observed lack of CDCA7 binding at the NCP in the previously tested mononucleosome (Nuc+64W; Fig. 3A), the HMZF domain was predicted to sterically clash with the nucleosome when the hemimethylated CpG was placed at this position (fig. S6B). Guided by the structure prediction, four additional locations of hemimethylated CpG in the Widom 601 nucleosome positioning sequence were tested for their recognition and binding by hCDCA7_264–371_ C339S (Fig. 4A, fig. S6C, and table S2) (*44*). Formation of the nucleosome-hCDCA7_264–371_ C339S complex was readily detected by EMSA for mononucleosomes where the structural model predicted successful binding (Nuc-58W; Nuc-58C). For those mononucleosomes where the structural model predicted that CDCA7 sterically clashes with the nucleosome (Nuc-62W; Nuc-64C), no clear nucleosome-hCDCA7_264–371_ C339S complex was detected (Fig. 4A and fig. S6C). Quantification of the nucleosome and free DNA signal detected by EMSA further shows that when the hemimethylated CpG is accessible within the NCP (Nuc-58W; Nuc-58C), the nucleosome, rather than free DNA, is preferentially bound by CDCA7, while the reverse is true when hemimethylated CpG is inaccessible on the nucleosome (Nuc-62W; Nuc-64C) (Fig. 4B). This suggests that CDCA7 binds more strongly to hemimethylated CpGs that are accessible on the NCP compared to those in free DNA (Fig. 4B). In contrast, nucleosomes remained unbound by the SRA domain regardless of the hemimethylated CpG position on the NCP (fig. S6D), whereas the SRA domain can bind to hemimethylated CpG in the linker DNA (fig. S6A). Together, these data demonstrate that the HMZF domain, but not the SRA domain, can sense hemimethylated CpGs within the outward facing major groove of the NCP.

We next conducted cryo-EM single-particle analysis on hCDCA7_264–371_ C339S in complex with the linker-free mononucleosome carrying a hemimethylated CpG at the NCP (Nuc-58W) (fig. S7 and tables S2 and S3). The initial cryo-EM map showed a density at the major groove of the NCP at the expected position of the hemimethylated CpG (fig. S7, A and B). 3D classification generated a cryo-EM map of 3.0-Å resolution for the NCP, and local refinement and local classification generated a ~4.0-Å resolution map for the extra cryo-EM density located at the hemimethylated CpG, which aligns well with the AF2-predicted structure of the HMZF domain of hCDCA7_264–371_ (Fig. 4C). This higher-resolution structure confirms that CDCA7 contacts the hemimethylated CpG at the major groove without eliciting any drastic distortions of the nucleosomal DNA. The R274 side chain forms a van der Waals interaction with the methyl group of the 5mC (-58W), while it also establishes a Hoogsteen-like pairing with the adjacent guanine (G; -57W) of the CpG dyad (Fig. 4D). R274 thus confers preferential binding of CDCA7 to a hemimethylated versus unmethylated CpG. Meanwhile, the side chain of Q275 is located within hydrogen bond distance of the cytosine (C; 57C) and guanine (G; 58C) of the unmethylated CpG dyad on the complementary strand. If this cytosine were to be methylated to form a symmetrically methylated CpG, it would sterically clash with the side chain of Q275 and consequently push R274 and destabilize its interaction with the original 5-methyl CpG (Fig. 4E). Q275 is highly conserved among CDCA7 homologs across eukaryotes (fig. S1) (*17*). Overall, the ICF-associated residue R274 appears to be one of the key residues that accounts for the highly selective binding specificity of CDCA7, whereas the ICF-associated residue R304, as well as the evolutionarily conserved S330 residue, contact the DNA phosphate backbone adjacent to the 5mC (Fig. 4F and fig. S1). ICF mutations R274C/H or R304H are predicted to disrupt CDCA7-DNA binding, while the third ICF-associated residue, G294, is positioned such that its ICF mutation (G294V) is predicted to disrupt the coordination of a zinc ion and negatively affect the overall structure of the HMZF domain of CDCA7 (fig. S7C).

The obtained structure of CDCA7 bound to the NCP (Fig. 4C) is distinct from the provisional model generated from the lower resolution cryo-EM density at the linker DNA (figs. S5C and S7D). This may indicate that CDCA7 adopts different configurations when sensing hemimethylated CpG at the NCP compared to the linker DNA. It is also possible that the low resolution of the cryo-EM density precluded accurate structure determination of the linker-bound CDCA7. Whether or not CDCA7 can engage hemimethylated CpG in multiple binding modes remains a future subject of the study.

### Characterization of the HELLS-CDCA7 interaction interface

**Fig. 5.**
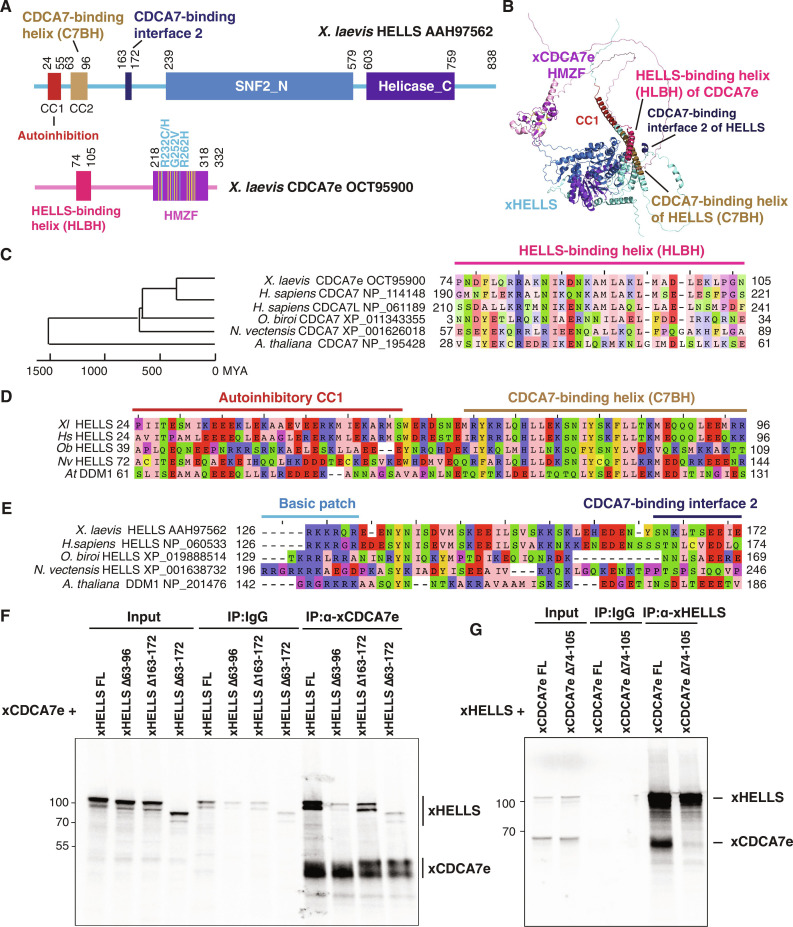
Identification of HELLS-CDCA7 interaction interface. (**A**) Schematics of *X. laevis* HELLS and CDCA7e. Positions of the signature 11 conserved cysteine residues and 3 ICF disease–associated mutations in CDCA7e are marked in yellow and cyan, respectively. CC1 is a coiled-coil domain important for autoinhibition. (**B**) The best predicted structure model of *X. laevis* HELLS-CDCA7e complex by AF2. (**C**) Sequence alignment of the putative HELLS/DDM1-binding interface of CDCA7. (**D**) Sequence alignment of the putative CDCA7-binding interface 1 in HELLS/DDM1. (**E**) Sequence alignment of the putative CDCA7-binding interface 2 in HELLS. (**F**) Immunoprecipitation by control IgG or anti-CDCA7e antibodies from *Xenopus* egg extracts containing ^35^S-labeled wild-type or deletion mutant of *X. laevis* HELLS and CDCA7e. (**G**) Immunoprecipitation by control IgG or anti-HELLS antibody from *Xenopus* egg extracts containing ^35^S-labeled HELLS and wild-type or ∆74-105 deletion mutant of CDCA7e. Autoradiography is shown in (F) and (G).

Our previous coevolution analysis has shown that the evolutionary preservation of CDCA7 is tightly coupled to the presence of HELLS; while CDCA7 and HELLS were frequently lost from several eukaryote lineages, all the tested eukaryotic species that encode CDCA7 also have HELLS (*17*). As this suggests an evolutionarily conserved function involving both CDCA7 and HELLS, we reasoned that the HELLS-CDCA7 interaction interface is likely also conserved in these species. We used AF2 structure prediction of HELLS-CDCA7 complex using sequences of HELLS/DDM1 and CDCA7 homologs from diverse eukaryotic species to identify likely CDCA7-HELLS interaction domains. In all tested cases [*X. laevis* HELLS-CDCA7e, *Homo sapiens* HELLS-CDCA7 and HELLS-CDCA7L, *Ooceraea biroi* (clonal raider ant) HELLS-CDCA7, *N. vectensis* HELLS-CDCA7, and *A. thaliana* DDM1-CDCA7], AF2 predicted the interaction of an N-terminal alpha helix of CDCA7 (amino acids 74 to 105 of *X. laevis* CDCA7e) with an N-terminal alpha helix of HELLS/DDM1 (amino acids 63 to 96 of *X. laevis* HELLS), as well as multiple segments within the SNF2_N domain of HELLS/DDM1 (Fig. 5, A and B, and fig. S8). The N-terminal putative CDCA7-binding alpha helix of HELLS corresponds to the previously annotated CC2 (coiled-coil2) segment, while it has been reported that the deletion of the preceding CC1 activates human HELLS by releasing its autoinhibition (*45*). AF2 also predicted an additional shorter CDCA7-binding interface in *X. laevis* and *H. sapiens* HELLS (amino acids 163 to 172 in *X. laevis* HELLS) (Fig. 5, A and B, and fig. S8, A to D). The putative interacting alpha helices of CDCA7 and HELLS/DDM1 are evolutionarily conserved in divergent green plant and animal species (Fig. 5, C and D, and figs. S8, C to G, and S9), whereas sequence conservation of the second CDCA7-binding interface in HELLS is less clear (Fig. 5E).

To experimentally validate these HELLS-CDCA7–binding interfaces, ^35^S-labeled *X. laevis* HELLS or CDCA7e proteins with or without these segments were incubated with *Xenopus* egg extracts to allow for binding to endogenous HELLS/CDCA7e proteins. Coimmunoprecipitation experiments demonstrate that deleting the first predicted CDCA7-binding interface of HELLS (amino acids 63 to 96) abolished HELLS-CDCA7e interaction, whereas deleting the second interface of HELLS (amino acids 163 to 172) also reduced CDCA7e binding, albeit to a lesser extent (Fig. 5F). This result suggests that the N-terminal CC2 of HELLS acts as a critical CDCA7-binding interface. Conversely, deleting the predicted HELLS-binding interface in CDCA7e (amino acids 74 to 105) abolished HELLS interaction (Fig. 5G). The result was also confirmed by using full-length or truncated versions of recombinant 3xFLAG-tagged CDCA7e (fig. S10); all mutants lacking the N-terminal alpha helix abolished HELLS binding, whereas the N-terminal portion that includes this alpha helix but lacks the HMZF domain retains robust HELLS binding. Together these data support the AF2 predicted model in which CDCA7 and HELLS interact via their evolutionarily conserved N-terminal helices. We name these helices in CDCA7 and HELLS, respectively, HLBH (HELLS-binding helix) and C7BH (CDCA7-binding helix).

### CDCA7 recruits HELLS to hemimethylated DNA

**Fig. 6.**
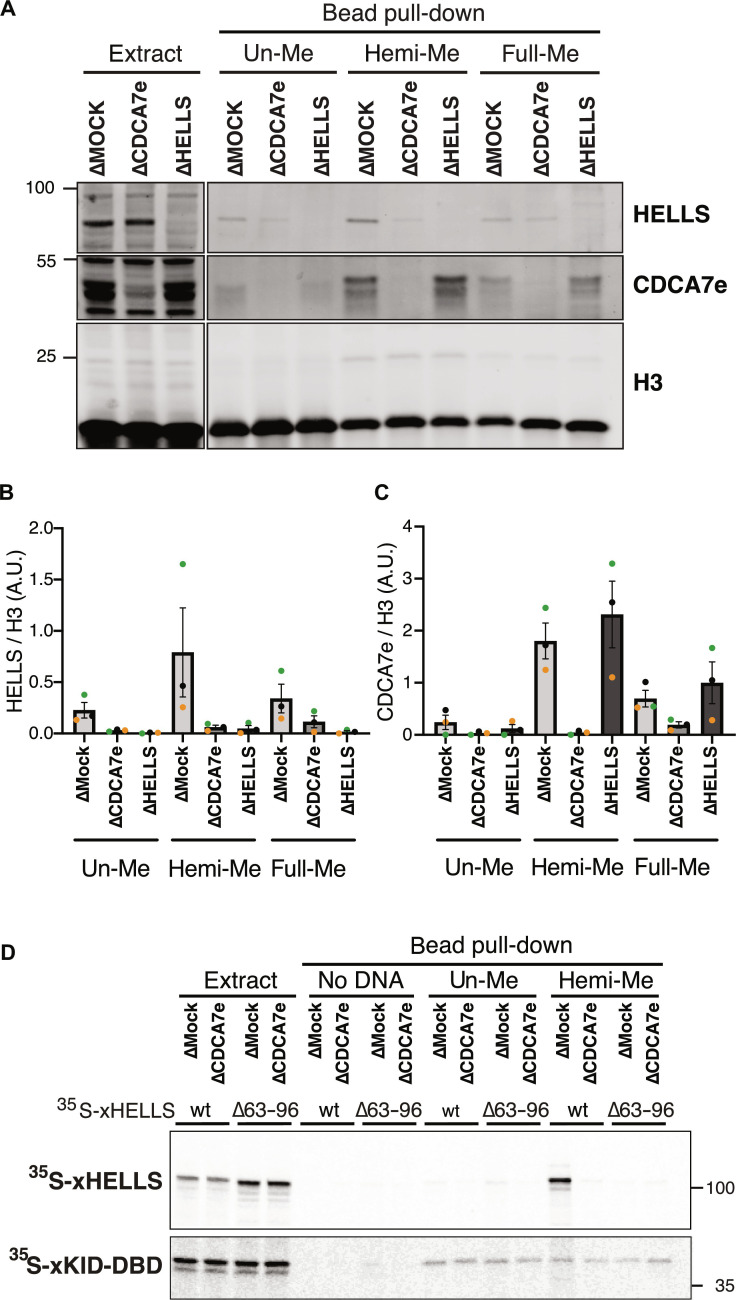
CDCA7 recruits HELLS to hemimethylated DNA. (**A**) Beads coated with unmethylated (un-Me), hemimethylated (hemi-Me), or fully methylated (full-Me) 3-kb DNA (pBluescript) were incubated with interphase *Xenopus* mock-depleted extract (∆MOCK), CDCA7e-depleted extract (∆CDCA7e), or HELLS-depleted extract (∆HELLS) for 10 min. Representative of *n* = 3 independent experiments. (**B**) Quantification of HELLS signal in Western blot analyses described in (A). HELLS signal at the DNA beads is normalized relative to the H3 signal. *n* = 3 (biological replicates). The means and SEM are shown. (**C**) Quantification of CDCA7e signal in the Western blot analyses described in (A). CDCA7e signal at the DNA beads is normalized relative to the H3 signal. Beads were isolated and analyzed by Western blotting. *n* = 3 (biological replicates). The means and SEM are shown. (**D**) ^35^S-labeled HELLS or HELLS ∆63-96 was incubated with beads coated with 200-bp unmethylated or hemimethylated DNA for 30 min in interphase *Xenopus* egg mock-depleted or CDCA7-depleted extracts. Beads were isolated, and associated ^35^S-labeled proteins were visualized by autoradiography. Nonspecific DNA binding protein xKid DNA binding domain (xKid-DBD) was used as a loading control.

The experiments above showed that CDCA7 directly binds to hemimethylated DNA (Figs. 1 to 4) and that both HELLS and CDCA7 are enriched on chromatin with hemimethylated DNA (fig. S2). To test whether HELLS accumulation onto hemimethylated DNA directly depends on CDCA7, unmethylated, hemimethylated, or fully methylated 3-kb DNA beads were incubated with mock -depleted (∆MOCK), CDCA7e-depleted (∆CDCA7e), or HELLS-depleted (∆HELLS) interphase egg extracts. HELLS reproducibly exhibited its highest binding at the hemimethylated substrate in mock-depleted extract (Fig. 6, A and B), and CDCA7e preferentially accumulated at hemimethylated DNA beads in mock- and HELLS-depleted extract (Fig. 6, A and C). Depletion of CDCA7e did not co-deplete HELLS from egg extracts but dramatically reduced the binding of HELLS to all DNA substrates (Fig. 6, A and B). Furthermore, when ^35^S-labeled HELLS was incubated with egg extracts, it preferentially bound to hemimethylated DNA over unmethylated DNA (Fig. 6D). This hemimethylated DNA–specific binding was abolished by CDCA7 depletion or deleting the CDCA7-binding helix from HELLS (C7BH: ∆63-96) (Fig. 5B). On the basis of these observations, we conclude that CDCA7 recruits HELLS to hemimethylated DNA.

### The role of HELLS and CDCA7 in UHRF1-mediated histone H3 ubiquitylation

Studies using ICF patient–derived cells and cell lines, as well as targeted depletion/knockout in culture cells, suggested that HELLS and CDCA7 are especially required for maintaining DNA methylation at heterochromatic, late-replicating regions (*6*, *27*, *37*, *46*). It was also suggested that HELLS/DDM1-dependent methylation is mediated by DNMT1/MET1 (plant DNMT1) (*14*, *47*). However, we did not detect any measurable impact of CDCA7e or HELLS depletion on maintenance DNA methylation of sperm or erythrocyte nuclei in *Xenopus* egg extracts as monitored by the incorporation of *S*-[methyl-^3^H]-adenosyl-l-methionine (fig. S11) (*24*). The apparent absence of a role for HELLS and CDCA7e in bulk maintenance DNA methylation could be explained by their function in replication-uncoupled maintenance methylation specifically, which is mediated by UHRF1-dependent H3 ubiquitylation (*25*). It has been shown in HeLa cells that HELLS facilitates UHRF1-mediated H3 ubiquitylation (*14*) and promotes the replication-uncoupled maintenance methylation at late-replicating regions (*27*).

**Fig. 7.**
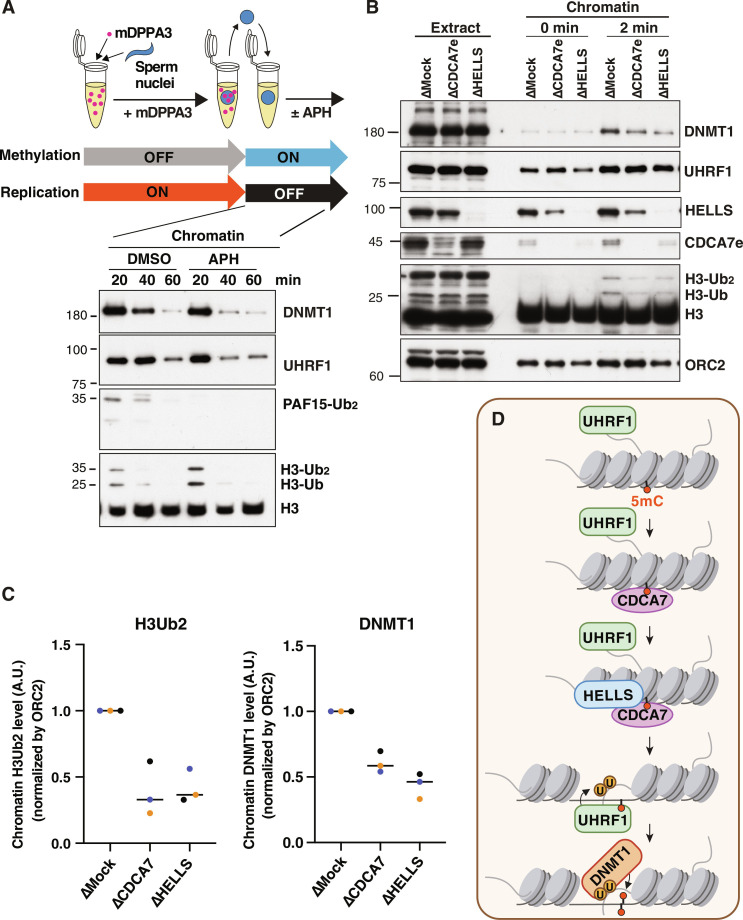
CDCA7e and HELLS regulate replication-uncoupled maintenance DNA methylation. (**A**) *Xenopus* sperm nuclei were incubated for 120 min in interphase *Xenopus* egg extract in the presence of 1.1 μM recombinant mDPPA3. Chromatin was isolated and reincubated in interphase egg extract in the presence or absence of 150 mM aphidicolin (APH). (**B**) Sperm nuclei were incubated for 120 min in mock-depleted extracts, CDCA7e-depleted or HELLS-depleted extracts supplemented with mDPPA3. Chromatin was isolated and reincubated in mock-depleted, CDCA7e-depleted or HELLS-depleted extracts in the presence of aphidicolin. Chromatin was then isolated at 0 and 2 min, and chromatin-bound proteins were analyzed by Western blotting using indicated antibodies (left). Representative of *n* = 3 independent experiments shown. (**C**) The intensity of dually monoubiquitylated H3 (H3Ub_2_) and DNMT1 signal relative to chromatin-bound ORC2 signal at 2 min was measured using ImageJ (*n* = 3). The means of the intensities of three independent experiment are shown as relative value (max = 1.0). Data points from each biological replicate are annotated in a unique color. (**D**) Schematic of the proposed function of CDCA7/HELLS in DNA methylation maintenance. A hemimethylated CpG in a nucleosome dense region is undetected by the SRA domain of UHRF1. CDCA7 detects the hemimethylated CpG on the nucleosome via the HMZF domain. CDCA7 recruits and activates HELLS, which unwraps DNA from the nucleosome to make the hemimethylated CpG accessible to the SRA domain of UHRF1, promoting its E3 ligase activity to ubiquitylate H3. DNMT1 activated by ubiquitylated H3 executes maintenance DNA methylation. DMSO, dimethyl sulfoxide.

Therefore, we next attempted to examine the potential role of CDCA7e and HELLS in H3 ubiquitylation on chromatin after DNA replication. For this purpose, we first induced the accumulation of hemimethylated CpG on sperm nuclei by replicating sperm chromatin in the presence of mDPPA3, which inhibits chromatin association of UHRF1 (Fig. 7A). The sperm nuclei containing hemimethylated DNA were subsequently transferred to fresh egg extracts with or without aphidicolin, which inhibits DNA replication. As expected, UHRF1 readily and transiently associated with these chromatin substrates after the transfer and promotes H3 ubiquitylation even in the presence of aphidicolin, demonstrating that UHRF1-mediated H3 ubiquitylation was uncoupled from DNA replication (Fig. 7A). To test the role of CDCA7 and HELLS in H3 ubiquitylation in the context of replication uncoupled maintenance methylation, we repeated this experiment using CDCA7- or HELLS-depleted extracts. In mock-depleted extract, CDCA7, HELLS, and UHRF1 were already detectable on the hemimethylated chromatin immediately upon nuclear transfer (0 min), whereas DNMT1 and ubiquitylated H3 emerged within 2 min. However, CDCA7 or HELLS depletion attenuated the appearance of chromatin-associated H3 ubiquitylation and DNMT1 without affecting the level of chromatin-bound UHRF1 (Fig. 7, B and C). CDCA7 depletion did not affect sperm DNA replication in egg extract (fig. S12), suggesting that the observed decrease in ubiquitylated H3 or DNMT1 accumulation was not caused by lower starting levels of hemimethylated DNA in the transferred sperm nuclei. Together, these results support the idea that CDCA7 recruits HELLS to hemimethylated chromatin to facilitate UHRF1-mediated replication-uncoupled H3 ubiquitylation, which in turn activates DNMT1 to promote maintenance DNA methylation.

## DISCUSSION

Among several SNF2-family ATPases that can remodel nucleosomes, HELLS/DDM1 plays a unique role in DNA methylation (*17*). It has also been reported that HELLS promotes replication-uncoupled maintenance DNA methylation by facilitating histone H3 ubiquitylation (*14*). Our present study revealed a previously missing molecular link between HELLS and the maintenance methylation pathway by identifying CDCA7 as a hemimethylated CpG sensor that recruits HELLS to hemimethylated DNA via its unique HMZF domain.

Although mutations of DNMT3B (ICF1), ZBTB24 (ICF2), CDCA7 (ICF3), and HELLS (ICF4) cause ICF syndrome, the genomic DNA methylation pattern in the de novo DNA methyltransferase–defective ICF1 patient cell lines is distinct from ICF2-4 cell lines, in which CpG-poor regions with heterochromatin features are particularly hypomethylated (*46*). In addition, coevolution analysis indicated that CDCA7 and HELLS have stronger evolutionary links to DNMT1 than to DNMT3 (*17*). These observations suggested that CDCA7 and HELLS promote DNA methylation in a mechanism distinct from de novo DNA methylation, which is now consolidated by our demonstration that the CDCA7 HMZF domain specifically recognizes hemimethylated CpG, the substrate of the maintenance DNA methyltransferase DNMT1. ICF disease–associated mutations in CDCA7 abolish its hemimethylated DNA binding, supporting the functional importance of hemimethylation detection by CDCA7.

UHRF1, the critical activator of DNMT1, selectively recognizes hemimethylated CpG to initiate DNA methylation maintenance (*19*–*21*). However, since UHRF1 cannot bind hemimethylated CpG within the NCP (*34*), it remained unclear whether a specialized mechanism exists to detect this mark on the NCP. Our study now demonstrates that the HMZF domain can sense hemimethylated CpG within the NCP, depending on its position. Cryo-EM structural analysis of CDCA7 bound at a hemimethylated CpG does not indicate any notable distortions of the hemimethylated DNA, which is in stark contrast to the base-flipping induced by the SRA domain of UHRF1 (*19*–*21*). Instead, our structural model indicates that 5mC recognition by the HMZF domain is limited to interactions within the major groove of the DNA. This allows hemimethylated CpG detection even within the NCP if it is positioned in accessible major grooves. Although not all hemimethylated CpGs on the NCP are readily accessible by CDCA7, particularly in the context of strong nucleosome positioning sequences such as Widom 601, we anticipate that most native DNA sequences are flexible enough to shift the CpG into accessible positions. Residues Q275 and R274, the latter of which is mutated in patients with ICF3, are key to the hemimethylation selectivity of CDCA7, while ICF-associated residue R304 is important to mediate contact with the DNA backbone. The role of R274 in forming a van der Waals contact with 5mC and a hydrogen bond with the adjacent 3′ guanine mimics the mechanism by which other methyl-binding proteins, such as MeCP2, recognize mCpG in the major groove (*48*). As MeCP2 additionally coordinates water molecules in the major groove to enhance 5mC binding (*49*), similar coordination of water molecules by the HMZF domain may contribute to hemimethylation selectivity.

In a recently reported crystal structure of CDCA7-bound to non-B form DNA, the Q275 and R274 residues also contact a hemimethylated CpG dyad, although the precise mechanism of hemimethylation discrimination appears to be different between the two structural models (*50*), suggesting that the HMZF domain may exhibit hemimethylation selectivity in several different DNA contexts. The overall 3D configuration of the HMZF domain is notably similar between our AF2-instructed model and the crystal structure, validating the usage of the AF2 model in our cryo-EM structure analysis.

We found that CDCA7 and HELLS assist H3 ubiquitylation and subsequent DNMT1 recruitment in hemimethylated sperm chromatin, suggesting that HELLS supports maintenance DNA methylation via UHRF1 activation at the chromatin, in line with a previously reported study (*14*). Although the complex regulation of the multi-domain UHRF1 protein is not yet fully understood, it has been shown that engagement of the SRA domain is important to mediate the allosteric activation of UHRF1 and target its E3 ligase activity to H3 (*51*, *52*). We envision that the recruitment and activation of HELLS to hemimethylated nucleosomes via CDCA7 unwraps DNA from the NCP and may additionally increase the accessibility of the histone H3 N-terminal tail otherwise associated with linker DNA (*53*), thereby promoting hemimethylated CpG binding of the SRA domain of UHRF1 and the subsequent activation of UHRF1 E3 ligase activity toward the H3 N-terminal tail (Fig. 7D) (*54*). In this way, hemimethylated CpG–binding by CDCA7 may promote methylation of DNA normally found within the NCP. We propose that the unique capability of CDCA7 to detect hemimethylated CpGs even in the context of the nucleosome sets the CDCA7-HELLS complex apart from other nucleosome remodelers and makes it uniquely suitable to promote DNA methylation. This may explain why CDCA7 and HELLS are particularly important in heterochromatin with low-methylated CpG density (*11*, *46*, *55*), where the chance that a hemimethylated CpG may be directly accessible to DNMT1 and activate its processive enzymatic activity is minimal (*56*, *57*).

Assisted by AF2 structural prediction, we demonstrated that two evolutionarily conserved alpha helices at the N-terminal regions of CDCA7 and HELLS are responsible for their interaction. It has been shown that HELLS on its own is catalytically inactive (*16*, *58*). Deleting the N-terminal alpha helix CC1 of human HELLS preceding the CDCA7-binding helix (C7BH/CC2) activates the ATPase and nucleosome remodeling activities of HELLS (*45*). Similarly, the N-terminal region of *Arabidopsis* DDM1 harboring CC1 and CC2 form an autoinhibitory (AutoN) domain (Fig. 5D) (*36*). Consistent with its proposed autoinhibitory function, the AF2 models predict that the highly acidic CC1 of *Arabidopsis* DDM1 associates with the basic cleft that captures DNA on the NCP (fig. S8, G to I) (*36*); this CC1 placement should interfere with DDM1 binding to the nucleosome. Intriguingly, the AF2 model predicts that the binding of CDCA7 is insufficient to affect CC1 association with the DNA binding cleft of DDM1 (fig. S8G). It is thus possible that the plant CDCA7 recruits DDM1 to hemimethylated DNA but is not essential for DDM1 activation, although it remains to be tested whether the plant CDCA7 binds DDM1. For animal HELLS homologs, CC1 and CC2 are predicted to form a long continuous helix (Fig. 5 and fig. S8, C to F), while the acidic feature of the autoinhibitory CC1 is evolutionarily conserved (Fig. 5D). Future studies are needed to test whether binding of CDCA7 activates HELLS/DDM1 by displacing the CC1 from the DNA binding cleft.

We note two limitations in this study. First, the capacity of CDCA7 to recognize hemimethylated DNA and promote maintenance methylation in the context of heterochromatic nucleosomes remains to be tested. However, since CDCA7 and HELLS are conserved in insects and other invertebrates, such as *N. vectensis*, where DNA methylation is largely associated with gene bodies and not with heterochromatic transposable elements (*59*, *60*), the role of CDCA7-HELLS is unlikely to be limited to heterochromatin. Although this study focused on the role of CDCA7 in maintenance methylation, it is possible that hemimethylated DNA sensing by CDCA7 also plays an important role in other processes, such as DNA repair, resolution of DNA-RNA hybrids, and macroH2A deposition (*37*, *61*–*65*). Second, although our data clearly show that CDCA7 selectively binds to DNA with a single hemimethylated CpG over unmethylated or symmetrically methylated CpG, further investigations are needed to test whether CDCA7 has more optimized substrates. The binding may be affected by DNA sequence, density, and spacing of hemimethylated CpG, or other modifications, such as 5-hydroxymethylcytosine. Paradoxically, we have previously shown that CDCA7 and HELLS abundantly associate with nucleosome arrays in *Xenopus* egg extracts in a manner independent of hemimethylated CpG or H3K9me3 (*16*), while the recombinant CDCA7 HMZF domain did not bind the mononucleosome if a hemimethylated CpG was placed at inaccessible positions (Fig. 3A and fig. S4A). The potential function of hemimethylation-independent CDCA7 binding to chromatin is a subject for future studies.

## MATERIALS AND METHODS

### *Xenopus* egg extracts

At the Rockefeller University, *X. laevis* was purchased from Nasco (female, LM00535MX) or Xenopus 1 (female, 4270; male, 4235); all vertebrate animal protocols (20031 and 23020) followed were approved by the Rockefeller University Institutional Animal Care and Use Committee. In Figs. 1 (A to C), 2 (C to F), 5 (F and G), and 6, and figs. S2 (A, D, and E) and S11, freshly prepared crude cytostatic factor (CSF) metaphase–arrested egg extracts were prepared as previously published (*66*). To prepare interphase extracts, 0.3 mM CaCl_2_ was added to CSF extract containing cycloheximide (250 ng/μl).

At the Institute of Medical Science, University of Tokyo, *X. laevis* was purchased from Kato-S Kagaku and handled according to the animal care regulations at the University of Tokyo. In Fig. 7 and figs. S2 (B and C), S10, and S12, clarified cytoplasmic extracts were used. Crude interphase egg extracts were prepared as described previously (*25*, *67*), supplemented with cycloheximide (50 μg/ml), cytochalasin B (20 μg/ml), 1 mM dithiothreitol (DTT), aprotinin (2 μg/ml), and leupeptin (5 μg/ml) and clarified by ultracentrifugation (Hitachi, CP100NX, P55ST2 swinging rotor) for 20 min at 48,400*g*. The cytoplasmic extracts were aliquoted, frozen in liquid nitrogen, and stored at −80°C. The clarified cytoplasmic extracts were supplemented with an energy regeneration system [2 mM adenosine triphosphate (ATP), 20 mM phosphocreatine, and creatine phosphokinase (5 μg/ml)].

### Chromatin isolation

*Xenopus* sperm nuclei (3000 to 4000 per μl) was added to interphase extract and incubated at 22°C. Extract was diluted 5- to 10-fold in chromatin purification buffer [(CPB): 50 mM KCl, 5 mM MgCl_2_, 2% sucrose, and 20 mM Hepes-KOH (pH 7.6)] supplemented with 0.1% Nonidet P-40 (NP-40). With the exception of Fig. 1A, CPB was additionally supplemented with 2 mM *N*-ethylmaleimide and 0.1 mM PR-619. Diluted extracts were layered onto a CPB-30% sucrose cushion and centrifuged at 15,000*g* for 10 min at 4°C. The chromatin pellet was recovered in 1× Laemmli sample buffer and boiled, and Western blotting was performed against the indicated proteins. For reincubation of hemimethylated chromatin, *Xenopus* sperm nuclei (3000 to 4000 per μl) were incubated with 80 μl mock-, CDCA7-, or HELLS-depleted extracts in the presence of 1.1 μM glutathione *S*-transferase (GST)–mDPPA3 for 120 min. Extracts were then diluted to 300 μl with CPB buffer and then added with 900 μl Cell lysis buffer (Wizard Genomic DNA Purification Kit, Promega). After 10 min incubation at room temperature (RT), (invert two to three times once during the incubation), chromatin was isolated by centrifugation at 16,500*g* for 20 s at RT in a fixed-angle rotor and washed with CPB buffer twice.

### Antibodies and Western blotting

*Xenopus* CDCA7e, HELLS, PAF15, DNMT1, and UHRF1 were detected with rabbit polyclonal antibodies previously described (*16*, *24*, *25*). Rabbit polyclonal histone H3 antibody (ab1791) was purchased from Abcam. Rabbit polyclonal histone H4 antibody (catalog no. 61521) was purchased from Active Motif. In Fig. 1A, fig. S2A, and Fig. 6A, antibodies were used in LI-COR Odyssey blocking buffer at the following dilutions: affinity purified anti-CDCA7e (2 μg/ml), affinity purified anti-HELLS (3.5 μg/ml), anti-UHRF1 serum (1:500), anti-H3 (1:1000), and anti-H4 (1:1000). Primary antibodies were detected with IRDye secondary antibodies (catalog no. 926-32211; catalog no. 926-68070, LI-COR BioSciences) and subsequently imaged and quantified on an Odyssey infrared imaging system. In fig. S2 (B and C), Fig. 7, and fig. S10, anti-DNMT1 (1:500) and anti-UHRF1 (1:500) sera were used in 5% milk in phosphate-buffered saline with 0.05% Tween 20 (PBS-T); anti-PAF15 (1:500), anti-CDCA7e (1:500), and anti-HELLS (1:500) sera were used in Sol 1 (Toboyo, Can Get Signal immunoreaction enhancer solution). Primary antibodies were detected with horseradish peroxidase (HRP)–conjugated secondary antibodies (rabbit IgG–, Protein A–, or mouse IgG–conjugated with HRP, Thermo Fisher Scientific) and enhanced chemiluminescence detection reagent (Amersham). After exposure to the wrapped membrane, x-ray film was developed. The intensity of each band was quantified using ImageJ by measuring background-subtracted regions of interest intensities.

### Immunodepletion

To immunodeplete CDCA7e or HELLS from extracts used for DNA beads pull-down experiments, 37.5 μg of affinity purified anti-CDCA7 or anti-HELLS antibodies was coupled to 150 μl of Protein A Dynabeads (Thermo Fisher Scientific) and used to deplete 100 μl of extract at 4°C for 45 min. To immunodeplete CDCA7e or HELLS from extract used for chromatin isolation experiments, 170 μl of antiserum was coupled to 40 μl of recombinant Protein A Sepharose (rPAS, GE Healthcare). Antibodies bound beads were washed extensively in CPB and supplemented with 4 μl of fresh rPAS. Beads were split into two portions, and 100 μl of extract was depleted in two rounds at 4°C, each for 1 hour. Mock depletion was performed using purified preimmune rabbit IgG (Sigma-Aldrich).

### Immunoprecipitations

For coimmunoprecipitation from *Xenopus* egg extracts in fig. S2 (D and E), anti-HELLS antibody (12.5 μg), or anti-UHRF1 serum (85 μl) were coupled to 50 μl of Protein A Dynabeads for 1 hour at RT. For coimmunoprecipitation from *Xenopus* egg extracts in Fig. 5 (F and G), anti-HELLS and anti-CDCA7e antibodies (25 μg) were coupled to 100 μl of Protein A Dynabeads for 1 hour at RT and cross-linked with Pierce BS_3_ (Thermo Fisher Scientific), following the manufacturer’s protocol. Antibody beads were washed extensively in sperm dilution buffer [5 mM Hepes, 100 mM KCl, 150 mM sucrose, and 1 mM MgCl_2_ (pH 8.0)]. UHRF1, HELLS, and CDCA7 mutants were cloned into pCS2 vector by Gibson assembly and radiolabeled with EasyTag l-[^35^S]-methionine (PerkinElmer) using the TnT Coupled Reticulocyte Lysate System (Promega) according to the manufacturer’s instructions. Immunoprecipitation was performed in interphase egg extracts (Fig. 5, F and G) supplemented with cycloheximide (250 ng/μl) and the following ratios of Tnt lysates: 7 μl of the indicated ^35^S-labeled HELLS mutants with 1 μl ^35^S-labeled xCDCA7e in 50 μl of extract (Fig. 5F), 7 μl of the indicated ^35^S-labeled CDCA7 mutants with 1 μl ^35^S-labeled HELLS in 50 μl of extract (Fig. 5G), 2 μl ^35^S-labeled UHRF1 and 2 μl ^35^S-labeled CDCA7 per 25 μl of extract (fig. S2D), and 3.5 μl ^35^S-labeled HELLS with 0.5 μl ^35^S-labeled UHRF1 per 25 μl of extract (fig. S2E). Extract was added to the beads and incubated on ice for 1 hour with flicking every 20 min. The extract was diluted with 10 volumes of CSF-XB [100 mM KCl, 1 mM MgCl_2_, 50 mM sucrose, 5 mM EGTA, and 10 mM Hepes (pH 8.0)], and beads were recovered on a magnet. Beads were washed and recovered three times with 150 μl of CSF-XB with 0.1% Triton X-100. Beads were resuspended in 1× Laemmli buffer and boiled, and supernatants were resolved by SDS–polyacrylamide gel electrophoresis (SDS-PAGE). Gels were fixed in fixative (1:2:7 glacial acetic acid:methanol:H_2_O), dried, and exposed on a PhosphorImager screen. Control immunoprecipitation was performed using purified preimmune rabbit IgG (Sigma-Aldrich).

### DNA pull-down assays

To generate unmethylated, fully methylated and hemimethylated biotinylated 54-bp DNA substrates, 54-bp DNA oligos listed in table S1 were annealed in a thermocycler and purified with size-exclusion chromatography, Superdex 200 Increase 10/300 GL (Cytiva). Unless otherwise indicated, the hemimethylated 54-bp DNA oligo contains the 5mCs in the forward strand. To generate biotinylated hemimethylated pBlueScript DNA substrates, a polymerase chain reaction (PCR)–linearized pBlueScript template was methylated by the CpG methyltransferase M.SssI according to manufacturer’s protocol (catalog no. EM0821, Thermo Fisher Scientific). DNA synthesis across the methylated linearized pBlueScript template was subsequently performed in Q5 High-Fidelity 2X Master Mix (New England Biolabs Inc.) using a 5′ biotinylated primer (5′-/5Biosg/CGTTCTTCGGGGCGAAAACTCTCAAGG -3′) purchased from Integrated DNA Technologies. The reaction mix was purified using the QIAquick PCR purification kit (QIAGEN), and the resultant hemimethylated DNA product was subsequently purified from the reaction mix by conjugation to streptavidin M280 Dynabeads (Invitrogen). For nonmethylated 3-kb DNA substrates, the above protocol was performed using unmethylated linearized pBlueScript DNA template during DNA synthesis. Fully methylated pBlueScript DNA substrates were generated by methylating the nonmethylated pBlueScript DNA substrates with CpG methyltransferase M.SssI (Thermo Fisher Scientific) before DNA-bead conjugation. Methylation status of all BlueScript DNA substrates was confirmed by restriction digest with BstUI (New England Biolabs Inc.). BlueScript DNA substrates were coupled to streptavidin beads at ~2 μg of DNA/5 μl of bead slurry in bead coupling buffer [50 mM tris-Cl, 0.25 mM EDTA, and 0.05% Triton X-100 (pH 8.0)] supplemented with 2.5% polyvinyl alcohol and 1.5 M NaCl for at least 2 hours at RT. Fifty-four–base pair DNA substrates (table S1) were conjugated to streptavidin M280 Dynabeads at ~500 ng of DNA/10 μl of bead slurry. Two hundred–base pair ultramers with Widom 601 nucleosome positioning sequence (table S1) (*44*) were purchased from Integrated DNA Technologies and conjugated to streptavidin M280 Dynabeads at ~1 μg of DNA/5 μl of bead slurry. After conjugation, DNA-streptavidin beads were collected and incubated in 50 mM tris-Cl, 0.25 mM EDTA, and 0.05% Triton X-100 with 1 mM biotin for at least 30 min. DNA beads were extensively washed in sperm dilution buffer [5 mM Hepes, 100 mM KCl, 150 mM sucrose, and 1 mM MgCl_2_ (pH 8.0)] before performing any pull-down assay. As boiled supernatants of cell lysates are known to prevent proteins from binding to beads nonspecifically (*68*), in vitro DNA pull-downs were performed in boiled and clarified extract supernatant where indicated (Fig. 2, D to F) (*68*). Boiled and clarified egg extract supernatant was prepared by boiling CSF extract for 15 min followed by ultracentrifugation for 30 min at 260,000*g*. Supernatant was aliquoted, frozen in liquid nitrogen, and stored at −80°C. All DNA pull-downs were performed at 20°C.

For DNA bead pull-downs analyzed by Western blot (Figs. 1A and 6A and fig. S2A), 5 μl of DNA-conjugated beads were incubated in 30 μl of interphase *Xenopus* egg extract, supplemented with cycloheximide (250 ng/μl) and 300 μM aphidicolin. After incubation, the beads were washed three times in bead wash buffer (10 mM K-Hepes, 50 mM sucrose, 1 mM MgCl_2_, 100 mM KCl, 0.5 mM tris(2-carboxyethyl)phosphine (TCEP), and 0.1% Triton X-100). Beads were resuspended in 1× Laemmli buffer and boiled, and supernatants were resolved by SDS-PAGE. Western blotting was performed against the indicated proteins.

To assess protein binding by autoradiography [Figs. 1D, 2 (C to F), and 5 (F and D), and fig. S2 (D and E)], indicated proteins were expressed and radiolabeled with EasyTag l-[^35^S]-methionine (PerkinElmer) using the TnT Coupled Reticulocyte Lysate System (Promega) according to the manufacturer’s instructions. The C-terminally GFP-tagged DNA binding domain of xKid (xKid-DBD, amino acids 544 to 651) was cloned into pCS2 vector by Gibson assembly. cDNA of CDCA7 homologs was purchased from GenScript (clone IDs OAb17308D; OSf02364; and OCr101632D) and cloned into pCS2 vector by Gibson assembly. The cDNA purchased for *N. vectensis* (OSf02364) encodes a truncated protein sequence (EDO33918.1) that corresponds to amino acids 157 to 313 of the full-length protein (XP_001626018.2).

To assess the recruitment of HELLS to hemimethylated DNA in egg extract using autoradiography (Fig. 6D), 5 μl of DNA-conjugated beads were incubated in 30 μl of interphase *Xenopus* egg extract supplemented with cycloheximide (250 ng/μl), 300 μM aphidicolin 3 μl of ^35^S-labeled HELLS, and 0.9 μl of ^35^S-labeled xKid-DBD per microliter of extract. To assess the in vitro binding of CDCA7e ICF mutants to hemimethylated DNA by autoradiography (Fig. 1D), 5 μl DNA beads were incubated in 20 μl of binding buffer [10 mM Hepes, 100 mM NaCl, 0.025% Triton X-100, and 0.25 mM TCEP (pH 7.8)] supplemented with 4 μl of ^35^S-labeled CDCA7 and 1 μl of ^35^S-labeled xKid. To assess binding of CDCA7 homologs, DNA bead pull-down was performed using 10 μl of DNA-conjugated beads incubated in 30 μl of interphase *Xenopus* egg extract (Fig. 2C) or boiled and clarified extract supernatant (Fig. 2, D to F), supplemented with 3 μl of ^35^S-labeled CDCA7 and 0.9 μl of ^35^S-labeled xKid. Beads were washed and recovered three times and boiled in 1× Laemmli buffer, and supernatants were resolved by SDS-PAGE. For DNA quantitation, SDS-PAGE gel was stained with 0.01% SYBR-Safe (Thermo Fisher Scientific) for 15 min before imaging. Gel was fixed in fixative (1:2:7 glacial acetic acid:methanol:H_2_O), dried, and exposed on a PhosphorImager screen.

### Detection of DNA methylation maintenance and DNA replication in *Xenopus* egg extract

DNA methylation of replicating sperm or erythrocyte nuclei in egg extract was assayed by the incorporation of ^3^H-SAM (S-[methyl-^3^H]-adenosyl-l-methionine; PerkinElmer, NET155H). Demembranated sperm nuclei were prepared as published previously (*69*). Erythrocyte nuclei were prepared from blood collected from dead adult male *X. laevis* frogs that were euthanized for testis dissection, following the protocol published previously (*70*), with the addition of an extra dounce homogenization step before pelleting the nuclei over the 1 M sucrose cushion. Erythrocyte nuclei were stored at −20°C in 50% glycerol STMN buffer [10 mM NaCl, 10 mM tris (pH 7.4), 3 mM MgCl_2_, and 0.5% NP-40]. Sperm or erythrocyte nuclei were replicated in cycling egg extract (3000 nuclei/μl extract) supplemented with cycloheximide (250 ng/μl) and 0.335 μM ^3^H-SAM (82.3 Ci/mmol) for 1 hour at 20°C. Replication was inhibited by the addition of 200 nM of recombinant GST–tagged nondegradable geminin (fig. S11, an expression plasmid provided by W. Matthew Michael) (*41*) or 500 nM of His6-geminin (fig. S2C, a gift from T. Takahashi). The reaction was stopped by the addition of 9 volumes of CPB. Genomic DNA was purified using a Wizard Genomic DNA Purification Kit (Promega) according to the manufacturer’s instructions. DNA pellets were resuspended in scintillation fluid (ScintiVerse; Thermo Fisher Scientific) and quantified using a liquid scintillation counter (PerkinElmer, Tri-Carb 2910 TR). To monitor DNA replication in egg extracts, [α-^32^P] dATP (3000 Ci/mmol, PerkinElmer) and sperm nuclei were added to interphase extracts and incubated at 22°C. At each time point, extracts were diluted in reaction stop solution (1% SDS and 40 mM EDTA) and treated with Proteinase K (NACALAI TESQUE Inc.) at 37°C. The solutions were spotted onto Whatman glass microfiber filters followed by 5% trichloroacetic acid containing 2% pyrophosphate. Filters were washed twice in ethanol and dried. The incorporation of radioactivity was counted in the scintillation cocktail.

### Protein purification

For 3xFLAG-tagged full-length mDPPA3 or xCDCA7e expression in insect cells, Baculoviruses were produced using a BestBac v-cath/chiA Deleted Baculovirus Cotransfection kit (Expression system) following the manufacturer’s instructions. Proteins were expressed in Sf9 insect cells by infection with viruses expressing 3xFLAG-tagged mDPPA3 or xCDCA7e for 72 hours at 27°C. Sf9 cells from a 750-ml culture were collected and lysed by resuspending them in 30 ml of lysis buffer [20 mM tris-HCl (pH 8.0), 100 mM KCl, 5 mM MgCl_2_, 10% glycerol, 1% NP-40, 1 mM DTT, leupeptin (5 μg/ml), aprotinin (2 μg/ml), trypsin inhibitor (20 μg/ml), and phenylmethylsulfonyl fluoride (100 μg/ml)], followed by incubation on ice for 10 min. A soluble fraction was obtained after centrifugation of the lysate at 15,000*g* for 15 min at 4°C. The soluble fraction was incubated for 4 hours at 4°C with 250 μl of anti-FLAG M2 affinity resin equilibrated with lysis buffer. The beads were collected and washed with 10 ml of wash buffer and then with 5 ml of EB [20 mM Hepes-KOH (pH 7.5), 100 mM KCl, and 5 mM MgCl_2_] containing 1 mM DTT. Each recombinant protein was eluted twice in 250 μl of EB containing 1 mM DTT and 3xFLAG peptide (250 μg/ml, Sigma-Aldrich). Eluates were pooled and concentrated using a Vivaspin 500 (GE Healthcare).

cDNA of human CDCA7 encoding residues 264 to 371, 235 to 340, and 264 to 340 were subcloned into modified pGEX4T-3 plasmid (Cytiva) engineered for N-terminal GST and a small ubiquitin-like modifier-1 (SUMO-1) fusion tag (pGEX-ST) (*71*). cDNA of human UHRF1 SRA domain encoding residues 390 to 680 (SRA 399 to 680) was subcloned into modified pGEX-ST plasmid. Protein was expressed in *Escherichia coli* strain Rosetta 2 (DE3) (Novagen) for the expression of human CDCA7_264–371_, CDCA7_235–340_, CDCA7_264–340_, and human SRA_390–680_. The cells were grown at 37°C in LB medium containing ampicillin (50 μg/ml) and chloramphenicol (34 μg/ml) until reaching on optical density of 0.7 at 660 nm and then cultured in 0.2 mM isopropyl-β-d-thiogalactopyranoside for 15 hours at 15°C. The cells were lysed by sonication in 40 mM tris-HCl (pH 8.0) buffer containing 300 mM NaCl, 0.1 mM DTT (or 0.5 mM TCEP for CDCA7 residues 235 to 340 and 264 to 340), 30 μM zinc acetate, 10% (w/v) glycerol, and a protease inhibitor cocktail (Nacalai). After removing the debris by centrifugation, the supernatant was loaded onto Glutathione Sepharose 4B (Cytiva). After GST-SUMO tag was removed by SUMO-specific protease, the sample was loaded onto HiTrap Heparin column (Cytiva). Last, the protein was further purified using size-exclusion chromatography Hiload 26/600 S75 (Cytiva).

### Electrophoresis mobility shift assay

Ten microliters of samples were incubated for 30 min at 4°C in a binding buffer [20 mM tris-HCl (pH 7.5) containing 150 mM NaCl, 1 mM DTT, 0.05% NP-40, and 10% (w/v) glycerol], and electrophoresis was performed using a 0.5 × tris-acetate buffer [20 mM tris-ascetic acid containing 0.5 mM EDTA) at constant current of 8 mA for 100 min in a cold room on a 7.5% polyacrylamide gel purchased from Wako (SuperSep, Wako). Equimolar excess (0.5, 1.0, and 2.0) of the CDCA7 264-371 or 0.77, 1.54, and 3.85 equimolar excess of 3xFLAG-xCDCA7WT or 3xFLAG-xCDCA7R232H were added to the sample solution including 0.5 μM hemi-, full- and un-methylated DNA (upper: 5′- CAGGCAATCXGGTAGATC, lower: 5′-GATCTACXGGATTGCCTG, where X indicates cytosine or 5mC, and the hemimethylated DNA substrate contains 5mC in the upper strand, GeneDesign Inc.). Equimolar excess (3.0, 5.0 and 10.0) of the CDCA7 264-340 and 235-340 were added to the sample solution including the 0.5 μM DNA.

For analyzing the interaction with reconstituted nucleosomes, 0.5, 1.0, 2.0, and 3.0 equimolar excess of the CDCA7 264-371 or SRA 399-680 were added to 0.1 μM nucleosomes in 10 μl of reaction solution [binding buffer: 20 mM tris-HCl (pH 7.5), 50 mM NaCl, 1 mM DTT, 10% Glycerol, and 0.05% NP-40], and electrophoresis was performed using a 0.5 × TBE buffer (45 mM tris-borate and 1 mM EDTA) at constant current of 10 mA for 95 min in a cold room on a 7.5% polyacrylamide gel. To analyze the interactions, DNA was detected and analyzed by staining with GelRed (Wako) and the ChemiDoc XRS system (BIORAD), respectively.

### Nucleosome reconstruction

Recombinant human histone H2A, H2B, H3.1, and H4 proteins were produced in *E. coli* and purified using gel filtration chromatography and cation exchange chromatography as reported previously (*72*). The histone proteins were refolded into a histone octamer. All DNA including a single hemimethylated CpG were based on the Widom 601 nucleosome positioning sequence (*44*). For preparation of DNA with a hemimethylated CpG at the 5′-linker, the Widom 601 sequence was amplified using the primers (table S2, Eurofins Genomics). For preparation of DNA with a hemimethylated site in the 3′-linker and nucleosomal DNA, the Widom 601 sequence was amplified with BsmBI site at the 3′-region and digested by BsmBI (table S2). The fragment was ligated with oligonucleotides including a single hemimethylated CpG (table S2). DNAs with 5mC at positions of −58 and −62 on the Watson strand and −58 and −64 on the Crick strand of the Widom 601 sequence were amplified using primer containing 5mC (dyad base is position of ±0) (table S2). The DNAs were purified with anion-exchange chromatography, HiTrap Q HP (Cytiva). The histone octamers were reconstituted into nucleosome with purified DNAs by salt dialysis method, and the nucleosomes were purified with HiTrap Q HP. The purified nucleosomes were dialyzed against 20 mM tris-HCl buffer (pH 7.5) containing 1 mM DTT and 5% glycerol. The nucleosomes were frozen in liquid nitrogen and stored at −80°C.

### Cryo-EM data collection and data processing

Three microliters of the human CDCA7_264–371_ C339S in complex with the nucleosome harboring a single hemimethylated CpG, in which 5mC is positioned at +75 on Watson strand in the 3′-linker DNA (Nuc+75W) and −58 on Watson strand (Nuc-58W) of Widom 601 sequence, were applied onto the glow-discharged holey carbon grids (Quantifoil Cu R1.2/1.3, 300 mesh). The grids were plunge-frozen in liquid ethane using a Vitrobot Mark IV (Thermo Fisher Scientific). Parameters for plunge-freezing were set as follows: blotting time, 3 s; waiting time, 3 s; blotting force, −10; humidity, 100%; and chamber temperature, 4°C. Data were collected at RIKEN BDR on a 300-kV Krios G4 (Thermo Fisher Scientific) with a K3 direct electron detector (Gatan) with BioQuantum energy filter. A total of 4000 and 19,346 movies of the CDCA7:Nuc+75W and CDCD7:Nuc-58W complexes were recorded at a nominal magnification of ×105,000 with a pixel size of 0.83 Å, in a total exposure of 60.7 and 51.4 e^−^/Å^2^ per 48 frames with an exposure time of 2.2 and 2.6 s, respectively. The data were automatically acquired by the image shift method of the EPU software (Thermo Fisher Scientific), with a defocus range of −0.8 to −1.6 μm.

### Data processing and model refinement

All data were processed using cryoSPARC v4.2.1 and v4.4.0 (*73*). The movie stacks of were motion corrected by Patch Motion Correction. The defocus values were estimated from the contrast transfer function (CTF) by Patch CTF Estimation. Micrographs of CDCA7:Nuc+75W under 8-Å CTF resolution were cut off by Curate Exposures. A total of 1,881,583 and 9,315,129 particles of CDCA7:Nuc+75W and CDCD7:Nuc-58W complexes were automatically picked using Blob Picker, respectively. Particles (1,583,471 and 8,009,770 of CDCA7:Nuc+75W and CDCD7:Nuc-58W, respectively) were extracted using binning state (3.31 Å per pixel), and these particles were subjected to 2D classifications. Particles were further curated by heterogeneous refinement using the maps derived from cryoSPARC Ab-Initio Reconstruction as the template.

In the process of CDCA7:Nuc+75W, the selected suitable class containing 854,921 particles were classified by several round of heterogeneous refinement. Last, 3D reconstruction using 672,791 particles was performed by nonuniform refinement, and a 2.9-Å resolution map was obtained according to the gold-standard Fourier shell correlation = 0.143 criterion. For further classification, 3D variability and 3D classification were conducted, obtaining the maps at 3.3-Å resolution, which includes the map corresponding to the linker DNA region. Nonuniform refinement was performed using the particles selected by 3D variability and 3D classification (82,876 and 94,542 particles, respectively) (*71*), and a 3.2-Å resolution map was obtained (fig. S4). The focused mask corresponding to linker DNA containing the hemimethylated CpG bound by hCDCA7 was created for local refinement. Local refinement improved the map at 4.83-Å resolution (154,998 particles) (fig. S5). The structure of nucleosome moiety was created using Protein Data Bank (PDB) ID: 3LZ0. Linker DNA was generated using program Coot (*74*), and the structure of the nucleosome combined with linker DNA was refined with program PHENIX (*75*). A structure model of human CDCA7_264–371_ was generated from AF2 (AF-Q9BWT1-F1). The model was manually fitted to the focused map, taking into account the surface potential of the protein and characteristic C-terminal α helix of hCDCA7.

In the process of CDCA7:Nuc-58W, the selected suitable class containing 1,745,728 particles were classified by several round of heterogeneous refinement, resulting in reconstruction of 3D model using 1,501,235 particles performed by nonuniform refinement. For further categorize the structure, 3D classification was conducted, obtaining the maps at 3.0-Å resolution using 257,342 particles, which include the map corresponding to CDCA7 bound to 5mC at the -58 position on the Watson strand of Widom 601 sequence. The structure of nucleosome and CDCA7 moiety was created using PDB ID: 3LZ0 and AF2 model (AF-Q9BWT1-F1). The models were manually fitted to the map using the program Coot, and the structure of CDCA7:Nuc-58W was refined with the program PHENIX using phenix.real_space_refine.

Details of the data processing are shown in figs. S4, S5, and S7, and table S3. The protein structures were visualized using Pymol (The PyMOL Molecular Graphics System, version 2.2, Schrödinger LLC.) and UCSF ChimeraX (version 1.5).

### Prediction of the interactions between HELLS and CDCA7

We collected protein sequences of HELLS and CDCA7 from five species, including *H. sapiens*, *X. laevis*, *O. biroi*, *N. vectensis*, and *A. thaliana*. We then ran AF2 (version 2.2.2) (*76*) to predict the interactions between HELLS and CDCA7 in the five different species. For each prediction, we selected the best model for further structural analysis. We implemented a cutoff distance of 5 Å between nonhydrogen atoms to extract the interface residues between HELLS and CDCA7. The same cutoff was also applied to compute the pDockQ (*77*) metric for each of the five predictions. A pDockQ of greater than 0.23 indicates an acceptable predicted model, while a pDockQ of greater than 0.5 indicates a confident predicted model (*77*). To evaluate the convergence of the interface, we used MAFFT (*78*) to align the HELLS and CDCA7 protein sequences, respectively. The sequence alignments were visualized using MVIEW (*79*). The protein structures were visualized using Pymol (The PyMOL Molecular Graphics System, version 2.1, Schrödinger LLC).

### Statistical analysis

Cryo-EM data collection statistics are available in table S3.

## References

[1] A. L. Mattei, N. Bailly, A. Meissner, DNA methylation: A historical perspective. Trends Genet. 38, 676–707 (2022). 35504755 10.1016/j.tig.2022.03.010

[2] M. V. C. Greenberg, D. Bourc'his, The diverse roles of DNA methylation in mammalian development and disease. Nat. Rev. Mol. Cell Biol. 20, 590–607 (2019). 31399642 10.1038/s41580-019-0159-6

[3] A. Nishiyama, M. Nakanishi, Navigating the DNA methylation landscape of cancer. Trends Genet. 37, 1012–1027 (2021). 34120771 10.1016/j.tig.2021.05.002

[4] M. Ehrlich, K. Jackson, C. Weemaes, Immunodeficiency, centromeric region instability, facial anomalies syndrome (ICF). Orphanet J. Rare Dis. 1, 2 (2006). 16722602 10.1186/1750-1172-1-2 PMC1459120

[5] M. Vukic, L. Daxinger, DNA methylation in disease: Immunodeficiency, centromeric instability, facial anomalies syndrome. Essays. Biochem. 63, 773–783 (2019). 31724723 10.1042/EBC20190035 PMC6923317

[6] P. E. Thijssen, Y. Ito, G. Grillo, J. Wang, G. Velasco, H. Nitta, M. Unoki, M. Yoshihara, M. Suyama, Y. Sun, R. J. Lemmers, J. C. de Greef, A. Gennery, P. Picco, B. Kloeckener-Gruissem, T. Gungor, I. Reisli, C. Picard, K. Kebaili, B. Roquelaure, T. Iwai, I. Kondo, T. Kubota, M. M. van Ostaijen-Ten Dam, M. J. van Tol, C. Weemaes, C. Francastel, S. M. van der Maarel, H. Sasaki, Mutations in CDCA7 and HELLS cause immunodeficiency-centromeric instability-facial anomalies syndrome. Nat. Commun. 6, 7870 (2015). 26216346 10.1038/ncomms8870 PMC4519989

[7] M. Unoki, Chromatin remodeling in replication-uncoupled maintenance DNA methylation and chromosome stability: Insights from ICF syndrome studies. Genes Cells 26, 349–359 (2021). 33960584 10.1111/gtc.12850 PMC9292322

[8] S. Hardikar, Z. Ying, Y. Zeng, H. Zhao, B. Liu, N. Veland, K. McBride, X. Cheng, T. Chen, The ZBTB24-CDCA7 axis regulates HELLS enrichment at centromeric satellite repeats to facilitate DNA methylation. Protein Cell 11, 214–218 (2020). 31970665 10.1007/s13238-019-00682-w PMC7026229

[9] M. Unoki, G. Velasco, S. Kori, K. Arita, Y. Daigaku, W. K. A. Yeung, A. Fujimoto, H. Ohashi, T. Kubota, K. Miyake, H. Sasaki, Novel compound heterozygous mutations in UHRF1 are associated with atypical immunodeficiency, centromeric instability and facial anomalies syndrome with distinctive genome-wide DNA hypomethylation. Hum. Mol. Genet. 32, 1439–1456 (2023). 36458887 10.1093/hmg/ddac291

[10] D. S. Dunican, H. A. Cruickshanks, M. Suzuki, C. A. Semple, T. Davey, R. J. Arceci, J. Greally, I. R. Adams, R. R. Meehan, Lsh regulates LTR retrotransposon repression independently of Dnmt3b function. Genome Biol. 14, R146 (2013). 24367978 10.1186/gb-2013-14-12-r146 PMC4054100

[11] W. Yu, C. McIntosh, R. Lister, I. Zhu, Y. Han, J. Ren, D. Landsman, E. Lee, V. Briones, M. Terashima, R. Leighty, J. R. Ecker, K. Muegge, Genome-wide DNA methylation patterns in LSH mutant reveals de-repression of repeat elements and redundant epigenetic silencing pathways. Genome Res. 24, 1613–1623 (2014). 25170028 10.1101/gr.172015.114 PMC4199375

[12] A. Vongs, T. Kakutani, R. A. Martienssen, E. J. Richards, Arabidopsis thaliana DNA methylation mutants. Science 260, 1926–1928 (1993). 8316832 10.1126/science.8316832

[13] K. Dennis, T. Fan, T. Geiman, Q. Yan, K. Muegge, Lsh, a member of the SNF2 family, is required for genome-wide methylation. Genes Dev. 15, 2940–2944 (2001). 11711429 10.1101/gad.929101 PMC312825

[14] M. Han, J. Li, Y. Cao, Y. Huang, W. Li, H. Zhu, Q. Zhao, J. J. Han, Q. Wu, J. Li, J. Feng, J. Wong, A role for LSH in facilitating DNA methylation by DNMT1 through enhancing UHRF1 chromatin association. Nucleic Acids Res. 48, 12116–12134 (2020). 33170271 10.1093/nar/gkaa1003 PMC7708066

[15] D. B. Lyons, D. Zilberman, DDM1 and Lsh remodelers allow methylation of DNA wrapped in nucleosomes. eLife 6, e30674 (2017). 29140247 10.7554/eLife.30674 PMC5728721

[16] C. Jenness, S. Giunta, M. M. Muller, H. Kimura, T. W. Muir, H. Funabiki, HELLS and CDCA7 comprise a bipartite nucleosome remodeling complex defective in ICF syndrome. Proc. Natl. Acad. Sci. U.S.A. 115, E876–E885 (2018). 29339483 10.1073/pnas.1717509115 PMC5798369

[17] H. Funabiki, I. E. Wassing, Q. Jia, J. D. Luo, T. Carroll, Coevolution of the CDCA7-HELLS ICF-related nucleosome remodeling complex and DNA methyltransferases. eLife 12, RP86721 (2023). 37769127 10.7554/eLife.86721 PMC10538959

[18] F. Lyko, The DNA methyltransferase family: A versatile toolkit for epigenetic regulation. Nat. Rev. Genet. 19, 81–92 (2018). 29033456 10.1038/nrg.2017.80

[19] K. Arita, M. Ariyoshi, H. Tochio, Y. Nakamura, M. Shirakawa, Recognition of hemi-methylated DNA by the SRA protein UHRF1 by a base-flipping mechanism. Nature 455, 818–821 (2008). 18772891 10.1038/nature07249

[20] G. V. Avvakumov, J. R. Walker, S. Xue, Y. Li, S. Duan, C. Bronner, C. H. Arrowsmith, S. Dhe-Paganon, Structural basis for recognition of hemi-methylated DNA by the SRA domain of human UHRF1. Nature 455, 822–825 (2008). 18772889 10.1038/nature07273

[21] H. Hashimoto, J. R. Horton, X. Zhang, M. Bostick, S. E. Jacobsen, X. Cheng, The SRA domain of UHRF1 flips 5-methylcytosine out of the DNA helix. Nature 455, 826–829 (2008). 18772888 10.1038/nature07280 PMC2602803

[22] M. Bostick, J. K. Kim, P. O. Esteve, A. Clark, S. Pradhan, S. E. Jacobsen, UHRF1 plays a role in maintaining DNA methylation in mammalian cells. Science 317, 1760–1764 (2007). 17673620 10.1126/science.1147939

[23] J. Sharif, M. Muto, S. Takebayashi, I. Suetake, A. Iwamatsu, T. A. Endo, J. Shinga, Y. Mizutani-Koseki, T. Toyoda, K. Okamura, S. Tajima, K. Mitsuya, M. Okano, H. Koseki, The SRA protein Np95 mediates epigenetic inheritance by recruiting Dnmt1 to methylated DNA. Nature 450, 908–912 (2007). 17994007 10.1038/nature06397

[24] A. Nishiyama, L. Yamaguchi, J. Sharif, Y. Johmura, T. Kawamura, K. Nakanishi, S. Shimamura, K. Arita, T. Kodama, F. Ishikawa, H. Koseki, M. Nakanishi, Uhrf1-dependent H3K23 ubiquitylation couples maintenance DNA methylation and replication. Nature 502, 249–253 (2013). 24013172 10.1038/nature12488

[25] A. Nishiyama, C. B. Mulholland, S. Bultmann, S. Kori, A. Endo, Y. Saeki, W. Qin, C. Trummer, Y. Chiba, H. Yokoyama, S. Kumamoto, T. Kawakami, H. Hojo, G. Nagae, H. Aburatani, K. Tanaka, K. Arita, H. Leonhardt, M. Nakanishi, Two distinct modes of DNMT1 recruitment ensure stable maintenance DNA methylation. Nat. Commun. 11, 1222 (2020). 32144273 10.1038/s41467-020-15006-4 PMC7060239

[26] S. Ishiyama, A. Nishiyama, Y. Saeki, K. Moritsugu, D. Morimoto, L. Yamaguchi, N. Arai, R. Matsumura, T. Kawakami, Y. Mishima, H. Hojo, S. Shimamura, F. Ishikawa, S. Tajima, K. Tanaka, M. Ariyoshi, M. Shirakawa, M. Ikeguchi, A. Kidera, I. Suetake, K. Arita, M. Nakanishi, Structure of the Dnmt1 reader module complexed with a unique two-mono-ubiquitin mark on histone H3 reveals the basis for DNA methylation maintenance. Mol. Cell 68, 350–360.e7 (2017). 29053958 10.1016/j.molcel.2017.09.037

[27] X. Ming, Z. Zhang, Z. Zou, C. Lv, Q. Dong, Q. He, Y. Yi, Y. Li, H. Wang, B. Zhu, Kinetics and mechanisms of mitotic inheritance of DNA methylation and their roles in aging-associated methylome deterioration. Cell Res. 30, 980–996 (2020). 32581343 10.1038/s41422-020-0359-9 PMC7785024

[28] A. Kikuchi, H. Onoda, K. Yamaguchi, S. Kori, S. Matsuzawa, Y. Chiba, S. Tanimoto, S. Yoshimi, H. Sato, A. Yamagata, M. Shirouzu, N. Adachi, J. Sharif, H. Koseki, A. Nishiyama, M. Nakanishi, P. A. Defossez, K. Arita, Structural basis for activation of DNMT1. Nat. Commun. 13, 7130 (2022). 36414620 10.1038/s41467-022-34779-4 PMC9681727

[29] C. Zierhut, C. Jenness, H. Kimura, H. Funabiki, Nucleosomal regulation of chromatin composition and nuclear assembly revealed by histone depletion. Nat. Struct. Mol. Biol. 21, 617–625 (2014). 24952593 10.1038/nsmb.2845 PMC4082469

[30] M. Felle, H. Hoffmeister, J. Rothammer, A. Fuchs, J. H. Exler, G. Langst, Nucleosomes protect DNA from DNA methylation in vivo and in vitro. Nucleic Acids Res. 39, 6956–6969 (2011). 21622955 10.1093/nar/gkr263 PMC3167622

[31] M. Okuwaki, A. Verreault, Maintenance DNA methylation of nucleosome core particles. J. Biol. Chem. 279, 2904–2912 (2004). 14578347 10.1074/jbc.M310111200

[32] A. K. Robertson, T. M. Geiman, U. T. Sankpal, G. L. Hager, K. D. Robertson, Effects of chromatin structure on the enzymatic and DNA binding functions of DNA methyltransferases DNMT1 and Dnmt3a in vitro. Biochem. Biophys. Res. Commun. 322, 110–118 (2004). 15313181 10.1016/j.bbrc.2004.07.083

[33] H. Takeshima, I. Suetake, H. Shimahara, K. Ura, S. Tate, S. Tajima, Distinct DNA methylation activity of Dnmt3a and Dnmt3b towards naked and nucleosomal DNA. J. Biochem. 139, 503–515 (2006). 16567415 10.1093/jb/mvj044

[34] Q. Zhao, J. Zhang, R. Chen, L. Wang, B. Li, H. Cheng, X. Duan, H. Zhu, W. Wei, J. Li, Q. Wu, J. D. Han, W. Yu, S. Gao, G. Li, J. Wong, Dissecting the precise role of H3K9 methylation in crosstalk with DNA maintenance methylation in mammals. Nat. Commun. 7, 12464 (2016). 27554592 10.1038/ncomms12464 PMC5426519

[35] J. Brzeski, A. Jerzmanowski, Deficient in DNA methylation 1 (DDM1) defines a novel family of chromatin-remodeling factors. J. Biol. Chem. 278, 823–828 (2003). 12403775 10.1074/jbc.M209260200

[36] S. C. Lee, D. W. Adams, J. J. Ipsaro, J. Cahn, J. Lynn, H. S. Kim, B. Berube, V. Major, J. P. Calarco, C. LeBlanc, S. Bhattacharjee, U. Ramu, D. Grimanelli, Y. Jacob, P. Voigt, L. Joshua-Tor, R. A. Martienssen, Chromatin remodeling of histone H3 variants by DDM1 underlies epigenetic inheritance of DNA methylation. Cell 186, 4100–4116.e15 (2023). 37643610 10.1016/j.cell.2023.08.001 PMC10529913

[37] M. Unoki, H. Funabiki, G. Velasco, C. Francastel, H. Sasaki, CDCA7 and HELLS mutations undermine nonhomologous end joining in centromeric instability syndrome. J. Clin. Invest. 129, 78–92 (2019). 30307408 10.1172/JCI99751 PMC6307953

[38] C. E. Shamu, A. W. Murray, Sister chromatid separation in frog egg extracts requires DNA topoisomerase II activity during anaphase. J. Cell Biol. 117, 921–934 (1992). 1315785 10.1083/jcb.117.5.921 PMC2289485

[39] C. B. Mulholland, A. Nishiyama, J. Ryan, R. Nakamura, M. Yigit, I. M. Gluck, C. Trummer, W. Qin, M. D. Bartoschek, F. R. Traube, E. Parsa, E. Ugur, M. Modic, A. Acharya, P. Stolz, C. Ziegenhain, M. Wierer, W. Enard, T. Carell, D. C. Lamb, H. Takeda, M. Nakanishi, S. Bultmann, H. Leonhardt, Recent evolution of a TET-controlled and DPPA3/STELLA-driven pathway of passive DNA demethylation in mammals. Nat. Commun. 11, 5972 (2020). 33235224 10.1038/s41467-020-19603-1 PMC7686362

[40] K. Hata, N. Kobayashi, K. Sugimura, W. Qin, D. Haxholli, Y. Chiba, S. Yoshimi, G. Hayashi, H. Onoda, T. Ikegami, C. B. Mulholland, A. Nishiyama, M. Nakanishi, H. Leonhardt, T. Konuma, K. Arita, Structural basis for the unique multifaceted interaction of DPPA3 with the UHRF1 PHD finger. Nucleic Acids Res. 50, 12527–12542 (2022). 36420895 10.1093/nar/gkac1082 PMC9757060

[41] J. A. Wohlschlegel, B. T. Dwyer, S. K. Dhar, C. Cvetic, J. C. Walter, A. Dutta, Inhibition of eukaryotic DNA replication by geminin binding to Cdt1. Science 290, 2309–2312 (2000). 11125146 10.1126/science.290.5500.2309

[42] J. Jumper, R. Evans, A. Pritzel, T. Green, M. Figurnov, O. Ronneberger, K. Tunyasuvunakool, R. Bates, A. Zidek, A. Potapenko, A. Bridgland, C. Meyer, S. A. A. Kohl, A. J. Ballard, A. Cowie, B. Romera-Paredes, S. Nikolov, R. Jain, J. Adler, T. Back, S. Petersen, D. Reiman, E. Clancy, M. Zielinski, M. Steinegger, M. Pacholska, T. Berghammer, S. Bodenstein, D. Silver, O. Vinyals, A. W. Senior, K. Kavukcuoglu, P. Kohli, D. Hassabis, Highly accurate protein structure prediction with AlphaFold. Nature 596, 583–589 (2021). 34265844 10.1038/s41586-021-03819-2 PMC8371605

[43] M. Mirdita, K. Schutze, Y. Moriwaki, L. Heo, S. Ovchinnikov, M. Steinegger, ColabFold: Making protein folding accessible to all. Nat. Methods 19, 679–682 (2022). 35637307 10.1038/s41592-022-01488-1 PMC9184281

[44] P. T. Lowary, J. Widom, New DNA sequence rules for high affinity binding to histone octamer and sequence-directed nucleosome positioning. J. Mol. Biol. 276, 19–42 (1998). 9514715 10.1006/jmbi.1997.1494

[45] W. Nartey, A. A. Goodarzi, G. J. Williams, Cryo-EM structure of DDM1-HELLS chimera bound to nucleosome reveals a mechanism of chromatin remodeling and disease regulation. bioRxiv. 2023.2008.2009.551721 [Preprint] (2023). doi: 10.1101/2023.08.09.551721.

[46] G. Velasco, G. Grillo, N. Touleimat, L. Ferry, I. Ivkovic, F. Ribierre, J. F. Deleuze, S. Chantalat, C. Picard, C. Francastel, Comparative methylome analysis of ICF patients identifies heterochromatin loci that require ZBTB24, CDCA7 and HELLS for their methylated state. Hum. Mol. Genet. 27, 2409–2424 (2018). 29659838 10.1093/hmg/ddy130

[47] A. Zemach, M. Y. Kim, P. H. Hsieh, D. Coleman-Derr, L. Eshed-Williams, K. Thao, S. L. Harmer, D. Zilberman, The Arabidopsis nucleosome remodeler DDM1 allows DNA methyltransferases to access H1-containing heterochromatin. Cell 153, 193–205 (2013). 23540698 10.1016/j.cell.2013.02.033 PMC4035305

[48] Y. Liu, X. Zhang, R. M. Blumenthal, X. Cheng, A common mode of recognition for methylated CpG. Trends Biochem. Sci. 38, 177–183 (2013). 23352388 10.1016/j.tibs.2012.12.005 PMC3608759

[49] K. L. Ho, I. W. McNae, L. Schmiedeberg, R. J. Klose, A. P. Bird, M. D. Walkinshaw, MeCP2 binding to DNA depends upon hydration at methyl-CpG. Mol. Cell 29, 525–531 (2008). 18313390 10.1016/j.molcel.2007.12.028

[50] S. Hardikar, R. Ren, Z. Ying, J. R. Horton, M. D. Bramble, B. Liu, Y. Lu, B. Liu, J. Dan, X. Zhang, X. Cheng, T. Chen, The ICF syndrome protein CDCA7 harbors a unique DNA-binding domain that recognizes a CpG dyad in the context of a non-B DNA. bioRxiv. 2023.2012.2015.571946 [Preprint] (2023). doi: 10.1101/2023.12.15.571946. PMC11343032 39178265

[51] J. S. Harrison, E. M. Cornett, D. Goldfarb, P. A. DaRosa, Z. M. Li, F. Yan, B. M. Dickson, A. H. Guo, D. V. Cantu, L. Kaustov, P. J. Brown, C. H. Arrowsmith, D. A. Erie, M. B. Major, R. E. Klevit, K. Krajewski, B. Kuhlman, B. D. Strahl, S. B. Rothbart, Hemi-methylated DNA regulates DNA methylation inheritance through allosteric activation of H3 ubiquitylation by UHRF1. eLife 5, e17101 (2016). 27595565 10.7554/eLife.17101 PMC5012860

[52] J. Fang, J. Cheng, J. Wang, Q. Zhang, M. Liu, R. Gong, P. Wang, X. Zhang, Y. Feng, W. Lan, Z. Gong, C. Tang, J. Wong, H. Yang, C. Cao, Y. Xu, Hemi-methylated DNA opens a closed conformation of UHRF1 to facilitate its histone recognition. Nat. Commun. 7, 11197 (2016). 27045799 10.1038/ncomms11197 PMC4822050

[53] A. Stutzer, S. Liokatis, A. Kiesel, D. Schwarzer, R. Sprangers, J. Soding, P. Selenko, W. Fischle, Modulations of DNA contacts by linker histones and post-translational modifications determine the mobility and modifiability of nucleosomal H3 tails. Mol. Cell 61, 247–259 (2016). 26778125 10.1016/j.molcel.2015.12.015

[54] R. M. Vaughan, B. M. Dickson, M. F. Whelihan, A. L. Johnstone, E. M. Cornett, M. A. Cheek, C. A. Ausherman, M. W. Cowles, Z. W. Sun, S. B. Rothbart, Chromatin structure and its chemical modifications regulate the ubiquitin ligase substrate selectivity of UHRF1. Proc. Natl. Acad. Sci. U.S.A. 115, 8775–8780 (2018). 30104358 10.1073/pnas.1806373115 PMC6126761

[55] M. Vukic, J. Chouaref, V. Della Chiara, S. Dogan, F. Ratner, J. Z. M. Hogenboom, T. A. Epp, K. Chawengsaksophak, K. K. D. Vonk, C. Breukel, Y. Ariyurek, D. S. L. Granado, S. L. Kloet, L. Daxinger, CDCA7-associated global aberrant DNA hypomethylation translates to localized, tissue-specific transcriptional responses. Sci. Adv. 10, eadk3384 (2024). 38335290 10.1126/sciadv.adk3384 PMC10857554

[56] A. Hermann, R. Goyal, A. Jeltsch, The Dnmt1 DNA-(cytosine-C5)-methyltransferase methylates DNA processively with high preference for hemimethylated target sites. J. Biol. Chem. 279, 48350–48359 (2004). 15339928 10.1074/jbc.M403427200

[57] G. Vilkaitis, I. Suetake, S. Klimasauskas, S. Tajima, Processive methylation of hemimethylated CpG sites by mouse Dnmt1 DNA methyltransferase. J. Biol. Chem. 280, 64–72 (2005). 15509558 10.1074/jbc.M411126200

[58] J. Burrage, A. Termanis, A. Geissner, K. Myant, K. Gordon, I. Stancheva, The SNF2 family ATPase LSH promotes phosphorylation of H2AX and efficient repair of DNA double-strand breaks in mammalian cells. J. Cell Sci. 125, 5524–5534 (2012). 22946062 10.1242/jcs.111252 PMC3561860

[59] A. Zemach, I. E. McDaniel, P. Silva, D. Zilberman, Genome-wide evolutionary analysis of eukaryotic DNA methylation. Science 328, 916–919 (2010). 20395474 10.1126/science.1186366

[60] S. Feng, S. J. Cokus, X. Zhang, P. Y. Chen, M. Bostick, M. G. Goll, J. Hetzel, J. Jain, S. H. Strauss, M. E. Halpern, C. Ukomadu, K. C. Sadler, S. Pradhan, M. Pellegrini, S. E. Jacobsen, Conservation and divergence of methylation patterning in plants and animals. Proc. Natl. Acad. Sci. U.S.A. 107, 8689–8694 (2010). 20395551 10.1073/pnas.1002720107 PMC2889301

[61] G. Kollarovic, C. E. Topping, E. P. Shaw, A. L. Chambers, The human HELLS chromatin remodelling protein promotes end resection to facilitate homologous recombination and contributes to DSB repair within heterochromatin. Nucleic Acids Res. 48, 1872–1885 (2020). 31802118 10.1093/nar/gkz1146 PMC7038987

[62] M. Unoki, J. Sharif, Y. Saito, G. Velasco, C. Francastel, H. Koseki, H. Sasaki, CDCA7 and HELLS suppress DNA:RNA hybrid-associated DNA damage at pericentromeric repeats. Sci. Rep. 10, 17865 (2020). 33082427 10.1038/s41598-020-74636-2 PMC7576824

[63] X. Xu, K. Ni, Y. He, J. Ren, C. Sun, Y. Liu, M. I. Aladjem, S. Burkett, R. Finney, X. Ding, S. K. Sharan, K. Muegge, The epigenetic regulator LSH maintains fork protection and genomic stability via MacroH2A deposition and RAD51 filament formation. Nat. Commun. 12, 3520 (2021). 34112784 10.1038/s41467-021-23809-2 PMC8192551

[64] J. Zhou, X. Lei, S. Shafiq, W. Zhang, Q. Li, K. Li, J. Zhu, Z. Dong, X. J. He, Q. Sun, DDM1-mediated R-loop resolution and H2A.Z exclusion facilitates heterochromatin formation in Arabidopsis. Sci. Adv. 9, eadg2699 (2023). 37566662 10.1126/sciadv.adg2699 PMC10421056

[65] A. Osakabe, B. Jamge, E. Axelsson, S. A. Montgomery, S. Akimcheva, A. L. Kuehn, R. Pisupati, Z. J. Lorkovic, R. Yelagandula, T. Kakutani, F. Berger, The chromatin remodeler DDM1 prevents transposon mobility through deposition of histone variant H2A.W. Nat. Cell Biol. 23, 391–400 (2021). 33833428 10.1038/s41556-021-00658-1

[66] A. W. Murray, Cell Cycle Extracts. Methods. Cell Biol. 36, 573–597 (1991). 1839804

[67] S. Kumamoto, A. Nishiyama, Y. Chiba, R. Miyashita, C. Konishi, Y. Azuma, M. Nakanishi, HPF1-dependent PARP activation promotes LIG3-XRCC1-mediated backup pathway of Okazaki fragment ligation. Nucleic Acids Res. 49, 5003–5016 (2021). 33872376 10.1093/nar/gkab269 PMC8136790

[68] K. Tsuboyama, T. Osaki, E. Matsuura-Suzuki, H. Kozuka-Hata, Y. Okada, M. Oyama, Y. Ikeuchi, S. Iwasaki, Y. Tomari, A widespread family of heat-resistant obscure (Hero) proteins protect against protein instability and aggregation. PLOS Biol. 18, e3000632 (2020). 32163402 10.1371/journal.pbio.3000632 PMC7067378

[69] R. Lebofsky, T. Takahashi, J. C. Walter, DNA replication in nucleus-free Xenopus egg extracts. Methods Mol. Biol. 521, 229–252 (2009). 19563110 10.1007/978-1-60327-815-7_13

[70] S. E. Humphries, D. Young, D. Carroll, Chromatin structure of the 5S ribonucleic acid genes of Xenopus laevis. Biochemistry 18, 3223–3231 (1979). 465464 10.1021/bi00582a006

[71] K. Arita, S. Isogai, T. Oda, M. Unoki, K. Sugita, N. Sekiyama, K. Kuwata, R. Hamamoto, H. Tochio, M. Sato, M. Ariyoshi, M. Shirakawa, Recognition of modification status on a histone H3 tail by linked histone reader modules of the epigenetic regulator UHRF1. Proc. Natl. Acad. Sci. U.S.A. 109, 12950–12955 (2012). 22837395 10.1073/pnas.1203701109 PMC3420164

[72] K. Mayanagi, K. Saikusa, N. Miyazaki, S. Akashi, K. Iwasaki, Y. Nishimura, K. Morikawa, Y. Tsunaka, Structural visualization of key steps in nucleosome reorganization by human FACT. Sci. Rep. 9, 10183 (2019). 31308435 10.1038/s41598-019-46617-7 PMC6629675

[73] A. Punjani, J. L. Rubinstein, D. J. Fleet, M. A. Brubaker, cryoSPARC: Algorithms for rapid unsupervised cryo-EM structure determination. Nat. Methods 14, 290–296 (2017). 28165473 10.1038/nmeth.4169

[74] P. Emsley, B. Lohkamp, W. G. Scott, K. Cowtan, Features and development of Coot. Acta Crystallogr. D Biol. Crystallogr. 66, 486–501 (2010). 20383002 10.1107/S0907444910007493 PMC2852313

[75] P. V. Afonine, R. W. Grosse-Kunstleve, N. Echols, J. J. Headd, N. W. Moriarty, M. Mustyakimov, T. C. Terwilliger, A. Urzhumtsev, P. H. Zwart, P. D. Adams, Towards automated crystallographic structure refinement with phenix.refine. Acta Crystallogr. D Biol. Crystallogr. 68, 352–367 (2012). 22505256 10.1107/S0907444912001308 PMC3322595

[76] R. Evans, M. O’Neill, A. Pritzel, N. Antropova, A. Senior, T. Green, A. Žídek, R. Bates, S. Blackwell, J. Yim, O. Ronneberger, S. Bodenstein, M. Zielinski, A. Bridgland, A. Potapenko, A. Cowie, K. Tunyasuvunakool, R. Jain, E. Clancy, P. Kohli, J. Jumper, D. Hassabis, Protein complex prediction with AlphaFold-Multimer. bioRxiv. 2021.2010.2004.463034 [Preprint] (2022). doi: 10.1101/2021.10.04.463034.

[77] P. Bryant, G. Pozzati, A. Elofsson, Improved prediction of protein-protein interactions using AlphaFold2. Nat. Commun. 13, 1265 (2022). 35273146 10.1038/s41467-022-28865-w PMC8913741

[78] K. Katoh, K. Misawa, K. Kuma, T. Miyata, MAFFT: A novel method for rapid multiple sequence alignment based on fast Fourier transform. Nucleic Acids Res. 30, 3059–3066 (2002). 12136088 10.1093/nar/gkf436 PMC135756

[79] N. P. Brown, C. Leroy, C. Sander, MView: A web-compatible database search or multiple alignment viewer. Bioinformatics 14, 380–381 (1998). 9632837 10.1093/bioinformatics/14.4.380

[80] H. Funabiki, A. W. Murray, The Xenopus chromokinesin Xkid is essential for metaphase chromosome alignment and must be degraded to allow anaphase chromosome movement. Cell 102, 411–424 (2000). 10966104 10.1016/s0092-8674(00)00047-7

