# Supplementary material for: CDCA7 is an evolutionarily conserved hemimethylated DNA sensor in eukaryotes
Source: Sci Adv. 2024 Aug 23;10(34):eadp5753. doi: 10.1126/sciadv.adp5753 (PMC11343034; doi:10.1126/sciadv.adp5753)
Supplement: Supplementary file 1 — Figs. S1 to S12 Tables S1 to S3 [file sciadv.adp5753_sm.pdf]

Science Advances
AAAS

Supplementary Materials for

CDCA7 is an evolutionarily conserved hemimethylated DNA sensor in eukaryotes

Isabel E. Wassing *et al.*

Corresponding author: Atsuya Nishiyama, uanishiyama@g.ecc.u-tokyo.ac.jp;
Kyohei Arita, aritak@yokohama-cu.ac.jp; Hironori Funabiki, funabih@rockefeller.edu

Sci. Adv. **10**, eadp5753 (2024)
DOI: 10.1126/sciadv.adp5753

This PDF file includes:

Figs. S1 to S12
Tables S1 to S3

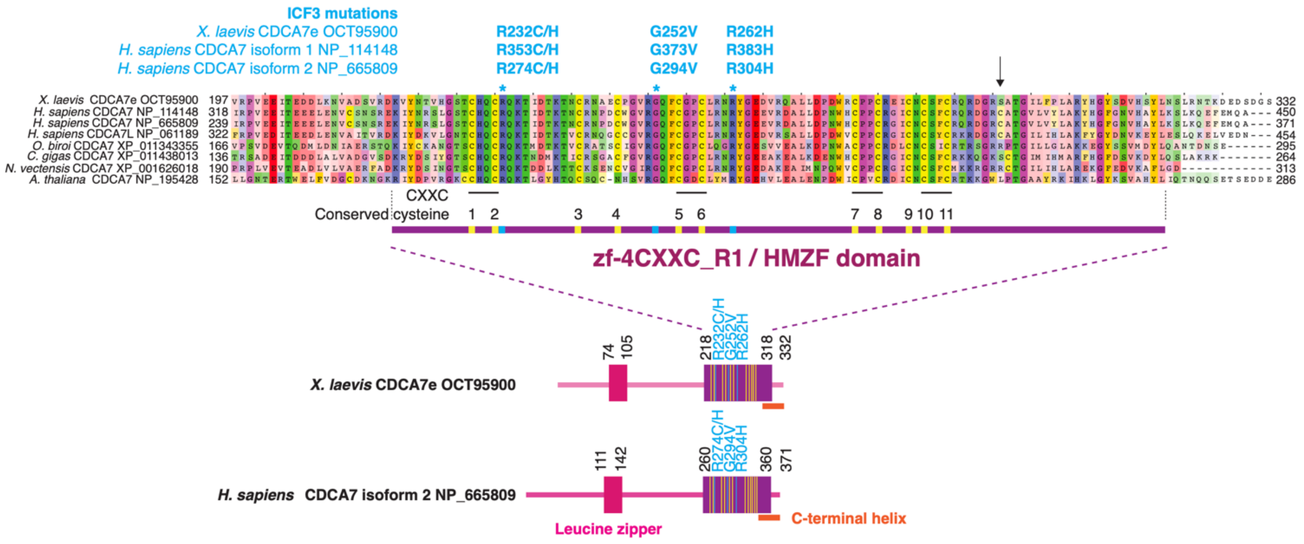

Fig. S1. Evolutionary conservation of the zf-4CXXC_R1 domain of CDCA7 homologs
ClustalW multi-sequence alignment of CDCA7 zf-4CXXC_R1 domain, also referred to as the hemimethylation-sensing zinc finger (HMZF) domain, characterized by eleven conserved cysteine (yellow) and three ICF3 patient-associated (cyan) residues. *X. laevis*, *Xenopus laevis* (African clawed frog); *H. sapiens*, *Homo sapiens* (human); *O. biroi*, *Ooceraea biroi* (clonal raider ant); *C. gigas, Crassostrea gigas* (Pacific oyster); *N. vectensis*, *Nematostella vectensis* (starlet sea anemone); *A. thaliana*, *Arabidopsis thaliana* (thale cress). Amino acid positions of ICF3 associated mutations in *X. laevis* CDCA7e, *H. sapiens* CDCA7 isoform 1 (NP_114148) and the shorter isoform 2 (NP_665809) are indicated. An arrow indicates the position of cysteine 339 of *H. sapiens* CDCA7 isoform 2, the site that was mutated to serine in Fig. 3, Fig. 4, fig. S3D, fig. S4 and fig. S5 and fig. S7. Schematics of the domain composition of *X. laevis* CDCA7e and *H. sapiens* CDCA7 isoform 2 are also shown.

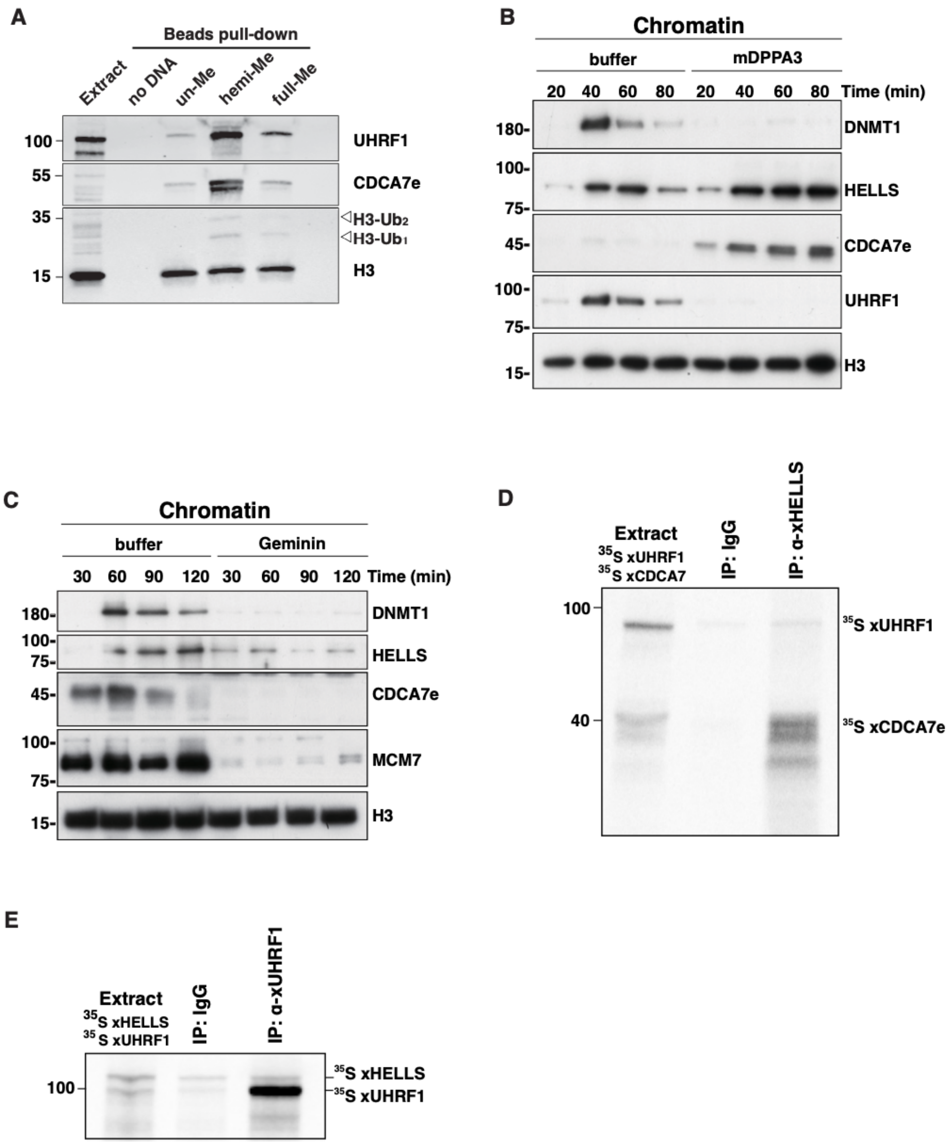

Fig. S2. HELLS and CDCA7 enrichment on hemi-methylated chromatin

(A) Magnetic beads coupled with 3 kb pBluescript DNA with unmethylated CpGs (un-Me), hemimethylated CpGs (hemi-Me), or fully methylated CpGs (full-Me), were incubated with interphase *Xenopus* egg extracts. Beads were collected after 60 min and analyzed by western blotting. **(B)** *X. laevis* sperm nuclei were isolated at indicated time points after incubation with interphase *Xenopus* egg extracts in the presence or absence of 0.5 μM mouse DPPA3 (mDPPA3), a protein that inhibits binding of UHRF1 and DNMT1 to chromatin. Chromatin-associated proteins were analyzed by western blotting. **(C)** *X. laevis* sperm nuclei were incubated with interphase *Xenopus* egg extracts in the presence or absence of 0.5 μM recombinant geminin. At each indicated time point, chromatin was isolated and analyzed by western blotting. **(D)** Immunoprecipitation by control IgG or anti-HELLS antibody from *Xenopus* egg extracts containing ^{35}S-labeled UHRF1 and ^{35}S-labeled CDCA7e. Representative of n=2 shown. **(E)** Immunoprecipitation by control IgG or anti-UHRF1 serum from *Xenopus* egg extracts containing ^{35}S-labeled HELLS and ^{35}S-labeled UHRF1. Autoradiography is shown in D and E.

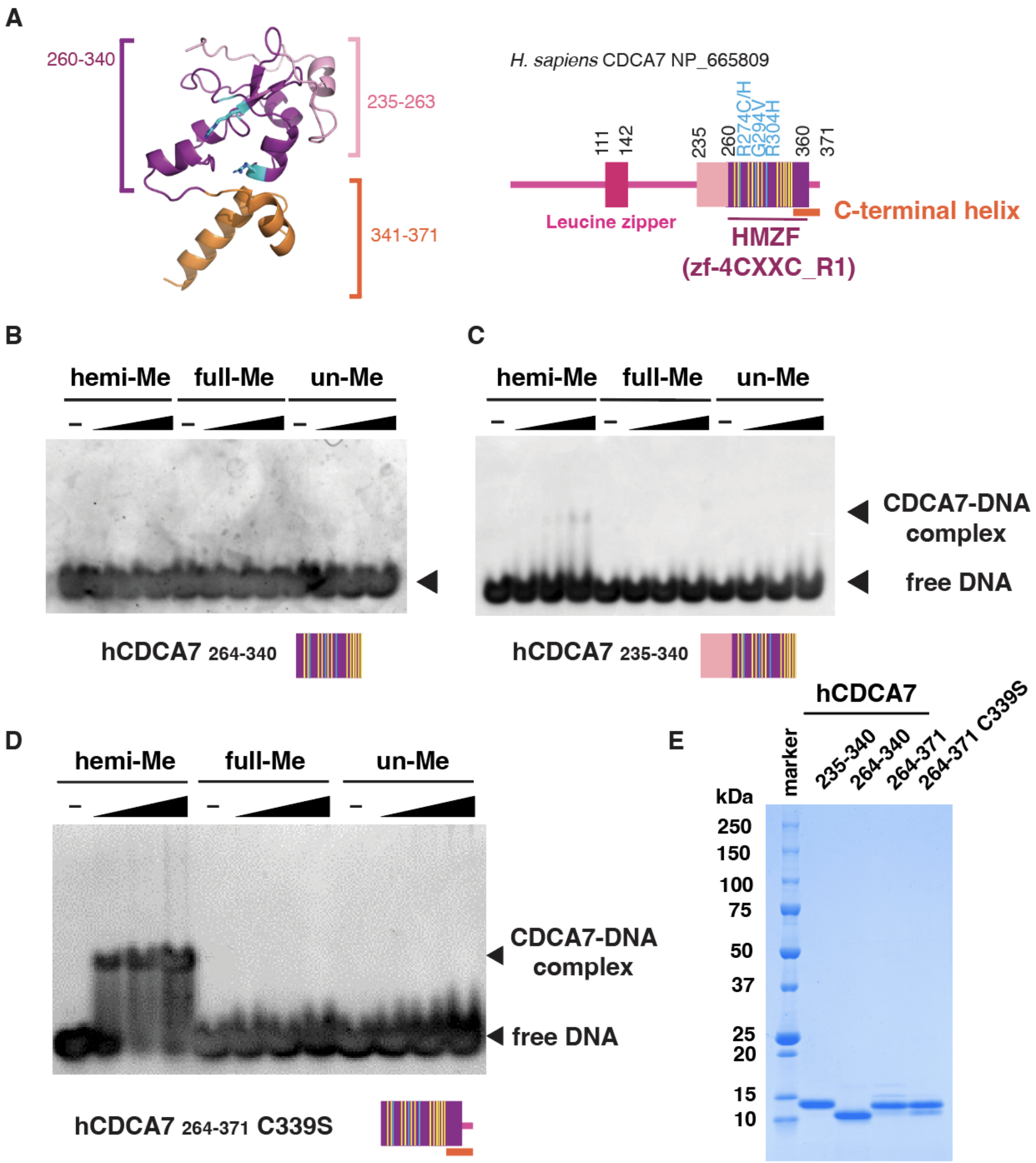

Fig. S3. Characterization of the minimum hemimethylated DNA-binding domain of human CDCA7

(**A**) AlphaFold2-modeled structure of the *H. sapiens* HMZF (zf-4CXXC_R1) domain and schematic of full-length *H. sapiens* CDCA7. Yellow lines indicate the position of conserved cysteine residues, three ICF3 patient-associated residues indicated in cyan. Orange bar indicates the conserved C-terminal helix. **(B-D)** Native gel electrophoresis mobility shift assay for detecting the interaction of hCDCA7 $_{264\text{-}340}$ **(B)** hCDCA7 $_{235\text{-}340}$ **(C)**, and hCDCA7 $_{264\text{-}371}$ C339S **(D)** with double stranded DNA oligonucleotides with a hemi-methylated (hemi-Me), fully-methylated (full-Me) or unmethylated (un-Me) CpG. **(E)** Coomassie-stained gel of the indicated purified hCDCA7 fragments.

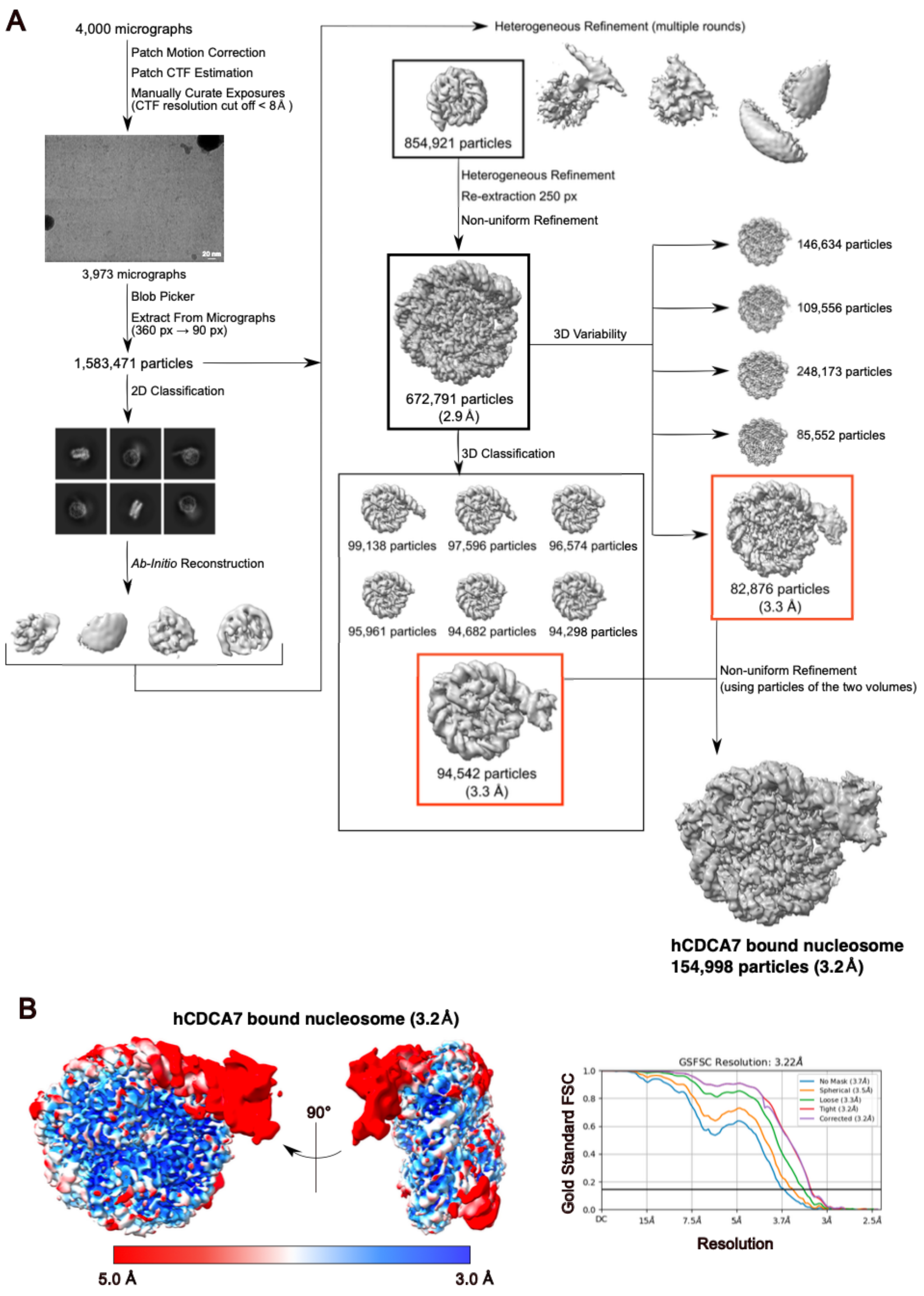

Fig. S4. Cryo-EM single particle analysis of hCDCA7 bound to linker DNA
(A) Cryo-EM data particle processing and refinement workflow of hCDCA7$_{264-371}$ C339S bound to Nuc+75W. **(B)** Local resolution of cryo-EM map of hCDCA7$_{264-371}$ C339S bound to Nuc+75W (left). Fourier shell correlation (FSC) curve of hCDCA7$_{264-371}$ C339S bound to linker DNA (right).

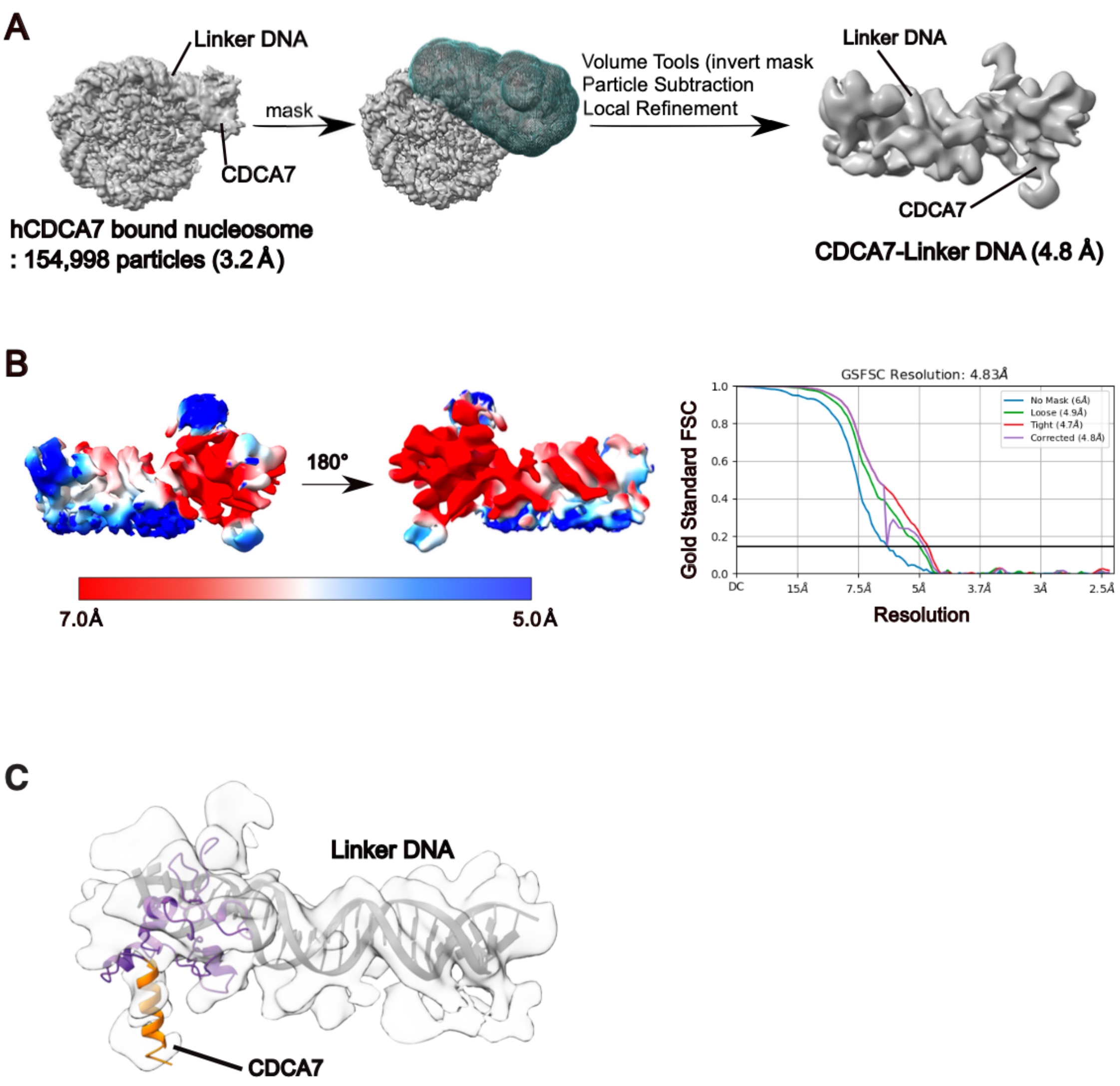

Fig. S5. Refinement workflow for cryo-EM map of linker DNA bound to hCDCA7 density
(A) Focused refinement of the linker DNA bound by hCDCA7$_{264-371}$ C339S moiety. Left figure shows the cryo-EM map of hCDCA7$_{264-371}$ C339S bound to nucleosome. The mask file is shown as a green mesh (center) covering the hCDCA7$_{264-371}$ C339S moiety bound to the linker DNA. The cryo-EM map corresponding to the hCDCA7$_{264-371}$ C339S moiety bound to the linker DNA was improved by local refinement at 4.83 Å resolution (right). **(B)** Local resolution of the hCDCA7$_{264-371}$ C339S moiety bound to linker DNA (left). FSC curve of hCDCA7$_{264-371}$ C339S bound to nucleosome (right). **(C)** Overlay of AF2 model of hCDCA7$_{264-371}$ C339S on the cryo-EM map. Conserved C-terminal helix indicated in orange.

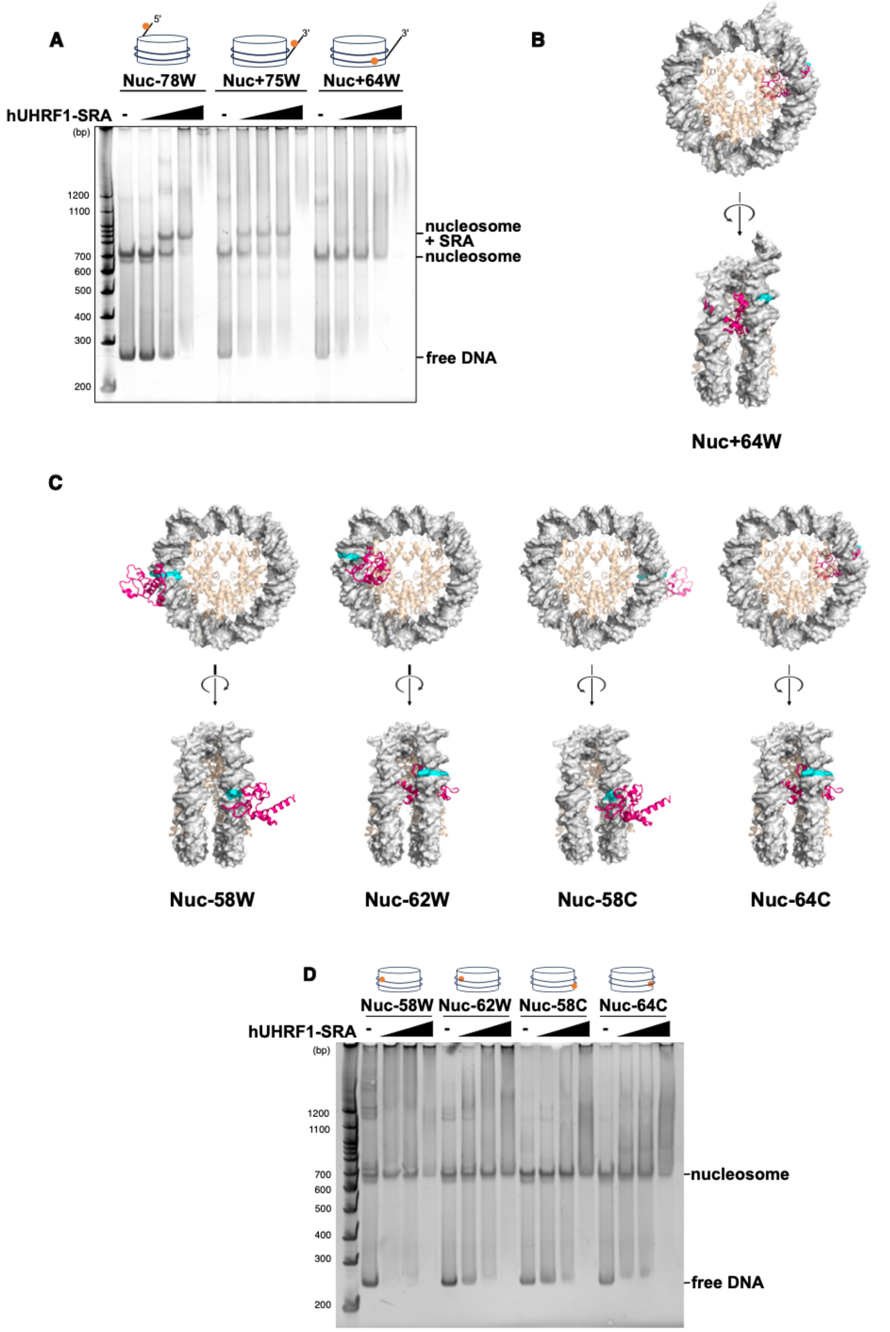

Fig. S6. Structure-guided predictions of CDCA7 binding at the nucleosome core particle
(A) Native gel electrophoresis mobility shift assay analyzing the interaction of human UHRF1 SRA domain with nucleosomes carrying hemimethylated CpG at the indicated positions (Table S2). **(B-C)** Predicted structure of hCDCA7$_{264\text{-}371}$ C339S bound to nucleosomes at the indicated positions, generated by superimposing the model structure of hCDCA7$_{264\text{-}371}$ C339S bound to the linker DNA. **(D)** Native gel electrophoresis mobility shift assay analyzing the interaction of human UHRF1 SRA domain with nucleosomes carrying hemimethylated CpG at the indicated positions (Table S2).

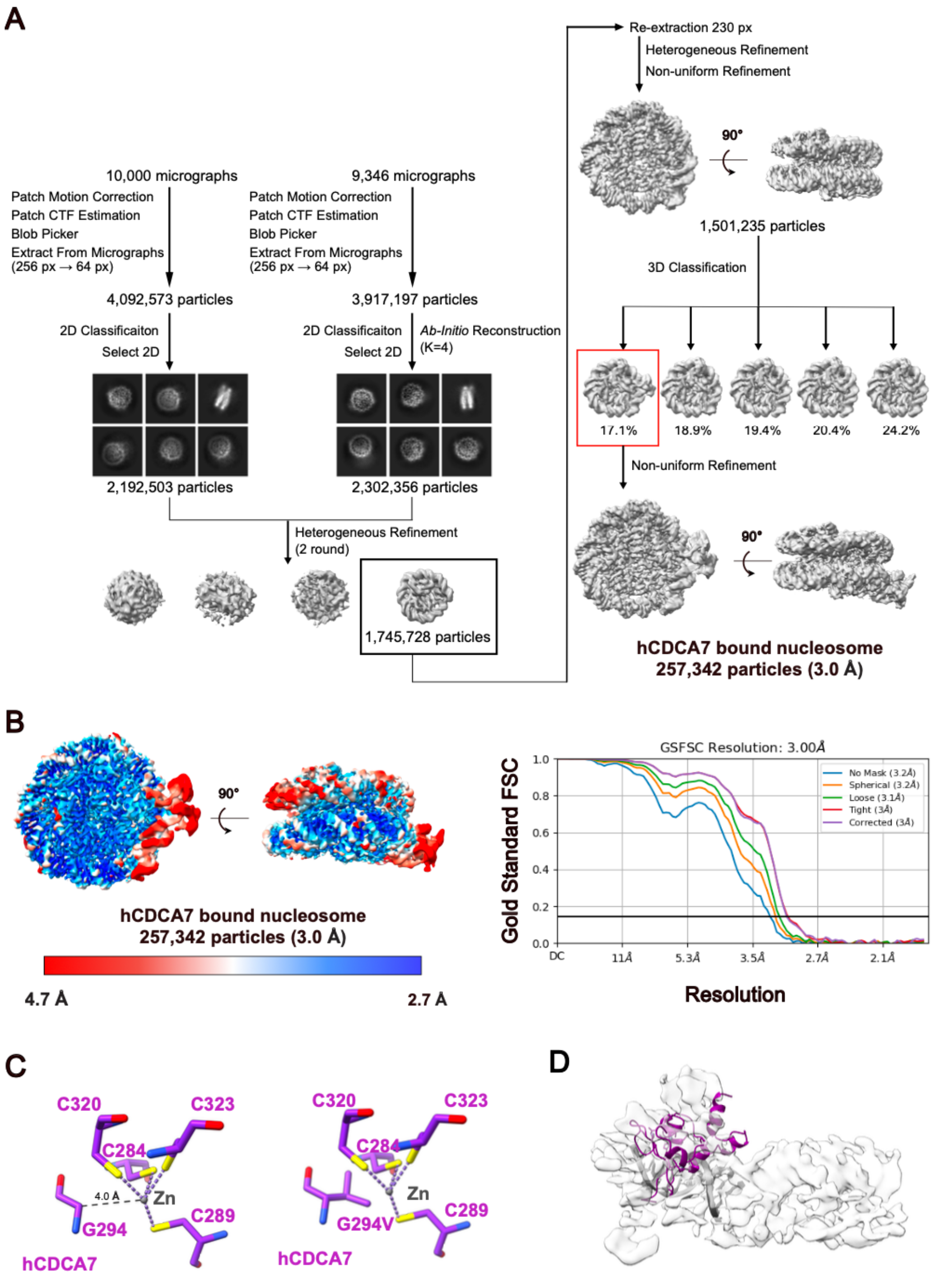

Fig. S7. Cryo-EM single particle analysis of hCDCA7 bound to nucleosome

(A) Cryo-EM data particle processing and refinement workflow of hCDCA7$_{264\text{-}371}$ C339S bound to the nucleosome (Nuc -58W). **(B)** Local resolution of cryo-EM map of hCDCA7$_{264\text{-}371}$ C339S bound to nucleosome (left). FSC curve of hCDCA7$_{264\text{-}371}$ C339S bound to nucleosome (right). **(C)** Predicted position of ICF disease-associated G294 relative to one of the coordinated zinc ions. **D)** hCDCA7$_{264\text{-}371}$ C339S structure model bound to Nuc-58W superimposed on cryo-EM density obtained for CDCA7 bound to Nuc+75W.

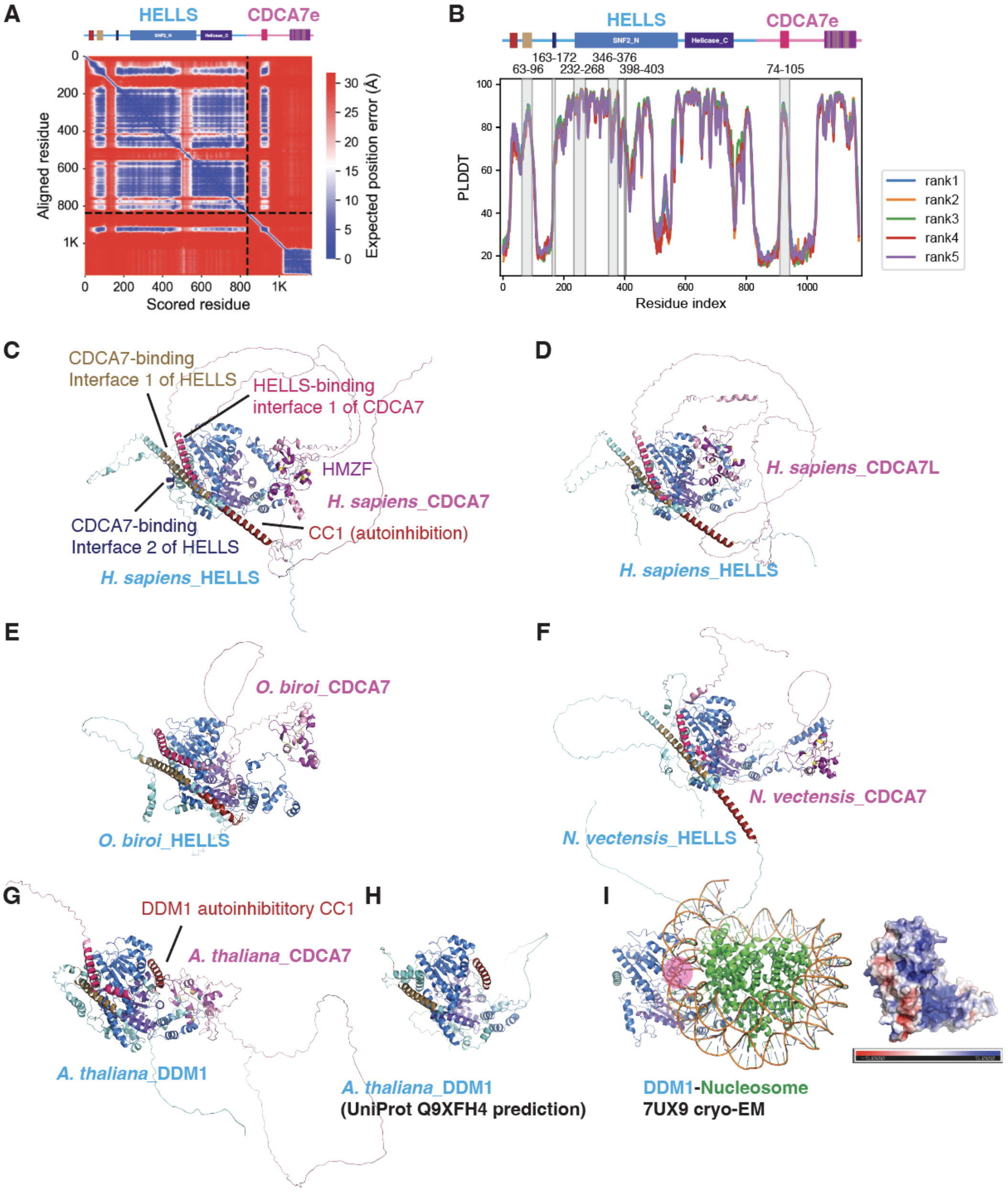

Fig. S8. AF2 structure prediction of the CDCA7-HELLS/DDM1 complex

(**A**, **B**) AF2 structure prediction analysis of *X. laevis* HELLS and CDCA7e. (**A**) The predicted aligned error map of the best model, with the minimum inter-chain predicted aligned error of 1.7 Å. (**B**) PLDDT scores of the top five predicted models, with the interface highlighted on top of the figure. The top five predictions were converged (region is shaded in gray), and the interface has relatively high PLDDT scores, with the average value of 63. (**C-H**) AF2 structure prediction of HELLS/DDM1 of indicated species in complex with CDCA7 and CDCA7 paralogs. (**I**) Left; atomic model of DDM1-nucleosome complex cryo-EM structure (7UX9). Right; surface electrostatic potential of DDM1. The DNA-binding positively charged groove, which is predicted to be occupied by the autoinhibitory CC by AlphaFold2 models, is marked with a pink circle.

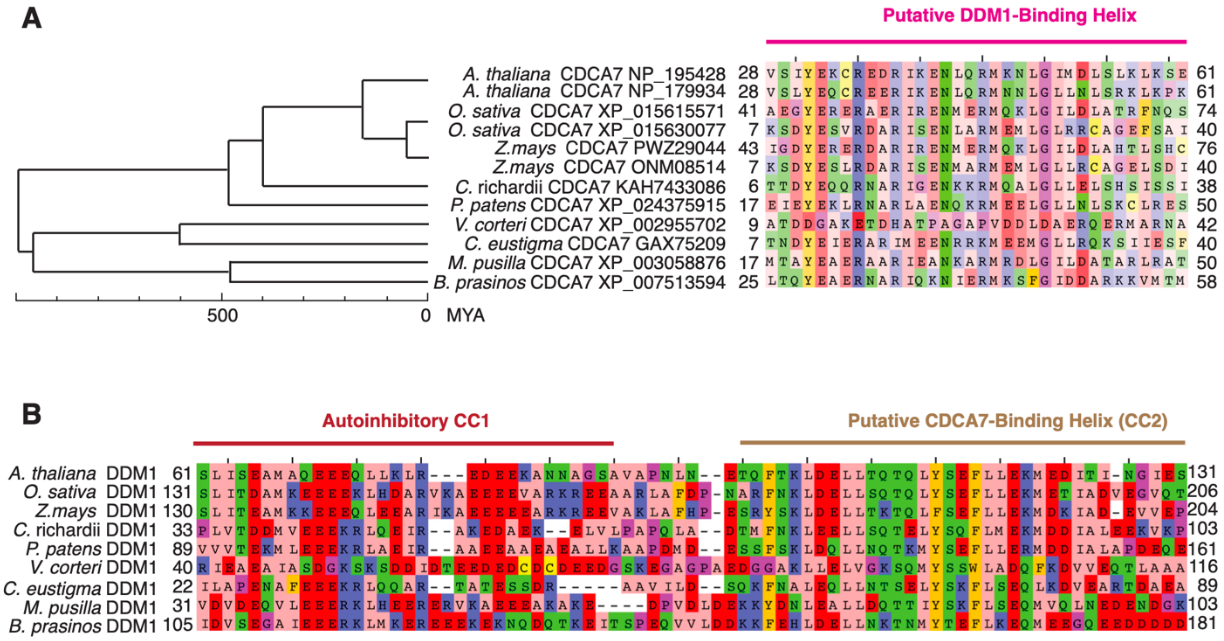

Fig. S9. Evolutionary conservation of putative DDM1-CDCA7 interaction interfaces in green plants

(A) Sequence alignment of putative DDM1-binding helix of CDCA7 homologs in green plants.

(B) Sequence alignment of the putative autoinhibitory CC1 and the CDCA7-binding CC2 of DDM1 homologs in green plants.

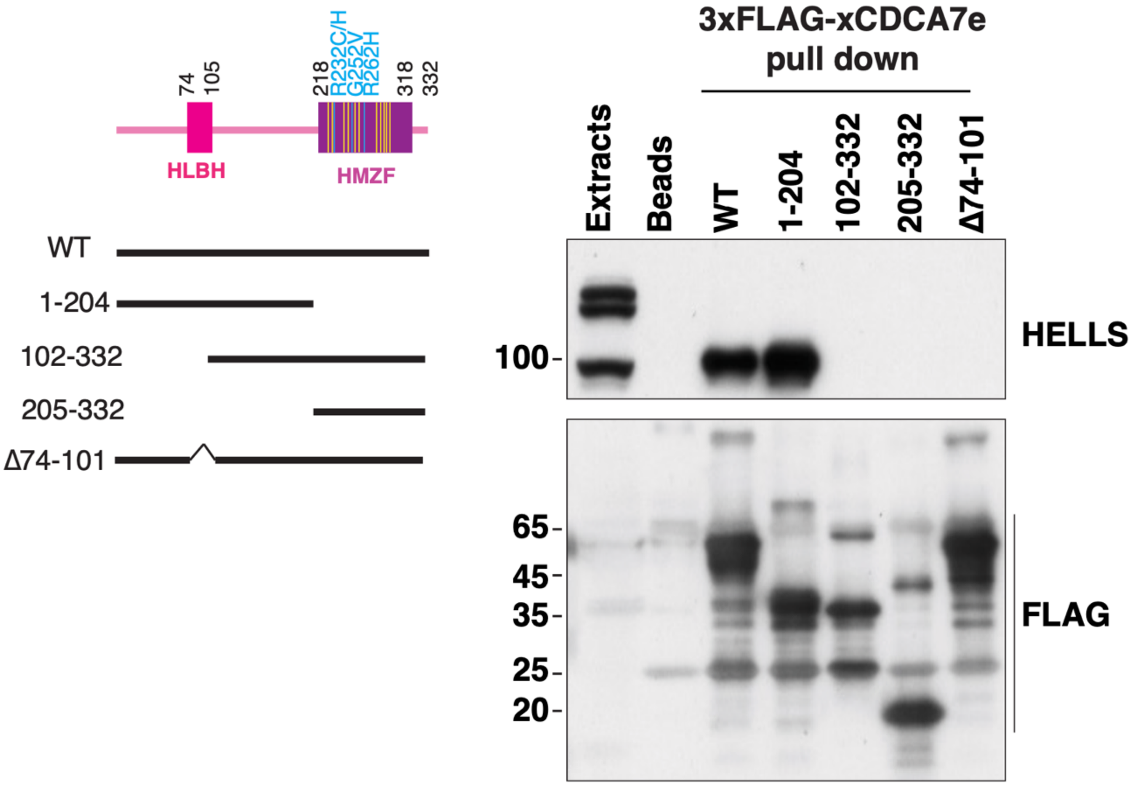

Fig. S10. N-terminal CDCA7 segment lacking the zf-4CXXC_R1 domain is sufficient for HELLS binding

Wildtype (WT) or truncated versions of recombinant FLAGx3-tagged *X. laevis* CDCA7e proteins were incubated with *Xenopus* egg extracts, followed by immunoprecipitation with anti-FLAG coupled beads. Isolated proteins were analyzed by western blotting using anti-HELLS and anti-FLAG antibodies.

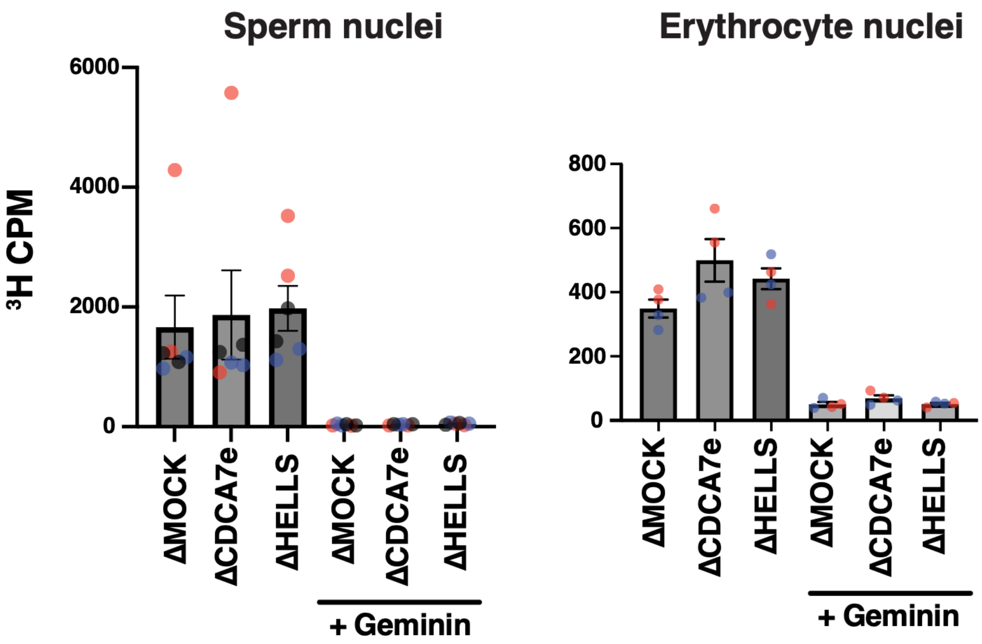

Fig. S11. CDCA7 and HELLS are not required for global maintenance DNA methylation in *Xenopus* egg extracts

X. laevis sperm nuclei **(A)** or erythrocyte nuclei **(B)** were incubated with egg extracts for 60 min with *S*-[methyl-^{3}H]-adenosyl-L-methionine with or without geminin, which inhibits DNA replication initiation. Radioactivity associated with chromosomal DNA is measured. Results include three biological replicates **(A)** or two biological replicates **(B)**, each of which includes two technical replicates (shown in the same color). Geminin (200 nM) effectively inhibited DNA incorporation of ^{3}H, demonstrating that DNA methylation of sperm chromatin depends on DNA replication.

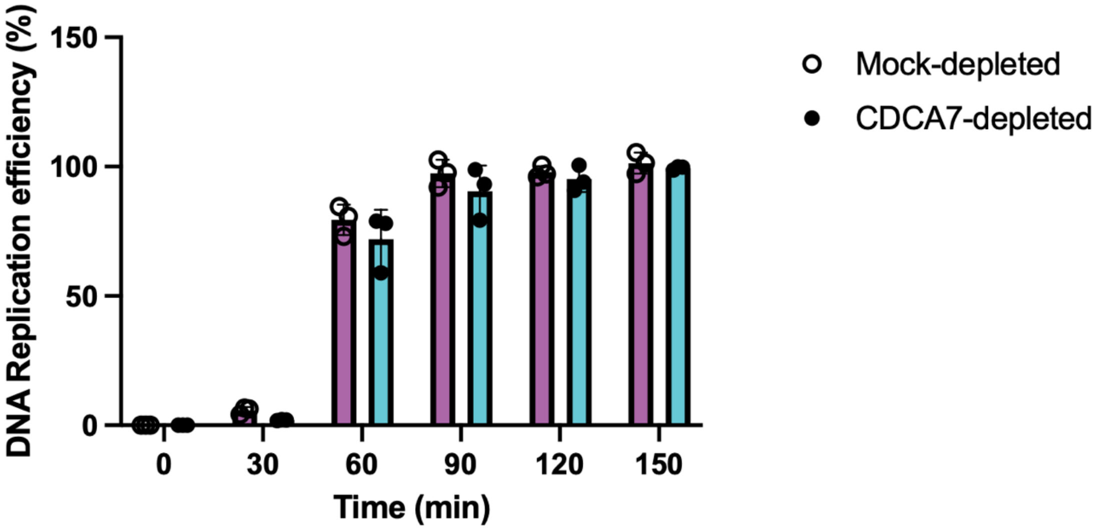

Fig. S12. CDCA7 depletion does not impact DNA replication in Xenopus egg extract
X. laevis sperm nuclei were incubated with mock- or CDCA7-depleted egg extracts for the indicated time points in the presence of [α-^{32}P] dATP. Radioactivity associated with chromosomal DNA replication is measured. Results include three biological replicates.

Table S1. DNA ultramer sequence used for DNA pull-downs

**Name**	**Sequence (sense strand)**
54 bp oligo non-Me Fw	GCGGGTGATGGACCCTATACGCGGCCGCCCTGGAGAA<br>TCCCGGTGCCGCGGCCG
54 bp oligo non-Me Rv	/5Biosg/CGGCCGCGGCACCGGGATTCTCCAGGGCGGC<br>CGCGTATAGGGTCCATCACCCGC
54 bp oligo Me Fw	GCGGGTGATGGACCCTATACGCGGCCGCCCTGGAGAA<br>TCC**M**GGTGC**M**G**M**GGC**M**G
54 bp oligo Me Rv	/5Biosg/**M**GGC**M**G**M**GGCAC**M**GGGATTCTCCAGGGCGG<br>CCGCGTATAGGGTCCATCACCCGC
200 bp unmethylated DNA Widom601	/5Biosg/TCGGGTTATGTGATGGACCCTATACGCGGGCG<br>CCCTGGAGAATCCTGCAGCCGAGGCCGCTCAATTGGT<br>CGTAGCAAGCTCTAGCACCGCTTAAACGCACGTACGC<br>GCTGTCCCCCGCGTTTTAACCGCCAAGGGGATTACTC<br>CCTAGTCTCCAGGCACGTGTCAGATATATACATCCTGT<br>GCATGTATTGAACAGCGAC
200 bp hemimethylated DNA Widom601	/5Biosg/T**M**GGGTTATGTGATGGACCCTATA**M**G**M**GGG**M**G<br>CCCTGGAGAATCCTGCAGC**M**GAGGC**M**GCTCAATTGGT<br>**M**GTAGCAAGCTCTAGCAC**M**GCTTAAA**M**GCA**M**GTA**M**G<br>**M**GCTGTCCCC**M**G**M**GTTTTAAC**M**GCCAAGGGGATTACT<br>CCCTAGTCTCCAGGCA**M**GTGTCAGATATATACATCCTG<br>TGCATGTATTGAACAG**M**GAC

*M:5-methylcytosine
**/5Biosg/: 5’ biotin modification

Table S2. DNA sequence used for nucleosome reconstruction

Name	Sequence (sense strand) and primers
5mC at -78* position on Watson strand in 5'-linker DNA (*Dyad is ±0 position)<br><br>**(Nuc-78W)**	ATCTGGGCC**M**GCCATATCAGAATCCCGGTGCCGAGGC<br>CGCTCAATTGGTCGTAGACAGCTCTAGCACCGCTTAAA<br>CGCACGTACGCGCTGTCCCCCGCGTTTTAACCGCCAA<br>GGGGATTACTCCCTAGTCTCCAGGCACGTGTCAGATAT<br>ATACATCGAT (160 bp)<br><br>**Primer**<br>Forward: 5'-<br>ATCTGGGCC**M**GCCATATCAGAATCCCGGTGCCGA<br>GGCCG<br>Reverse: 5'-ATCGATGTATATATCTGACACGTGC
5mC at +75 position on Watson strand in 3'-linker DNA<br><br>**(Nuc+75W)**	ATCAGAATCCCGGTGCCGAGGCCGCTCAATTGGTCGT<br>AGACAGCTCTAGCACCGCTTAAACGCACGTACGCGCT<br>GTCCCCCGCGTTTTAACCGCCAAGGGGATTACTCCCTA<br>GTCTCCAGGCACGTGTCAGATATATACATCGATCC**M**GC<br>AGGCC (157bp)<br><br>**Primer**<br>Forward: 5'- ATCAGAATCCCGGTGCCGAGGCCGC<br>Reverse: 5'- ATCCGTCTCCATCGATGTATATATC<br>**Oligo nucleotide**<br>Forward: 5'-CGATCC**M**GCAGGGCAG<br>Reverse: 5'-CTGCCCTGCGGG
5mC at +64 position on Watson strand in Widom601 sequence<br><br>**(Nuc+64W)**	ATCAGAATCCCGGTGCCGAGGCCGCTCAATTGGTCGT<br>AGACAGCTCTAGCACCGCTTAAACGCACGTACGCGCT<br>GTCCCCCGCGTTTTAACCGCCAAGGGGATTACTCCCTA<br>GTCTCCAGGCACGTGTCAGATATA**M**GCATCGATGCAG<br>G (150 bp)<br><br>**Primer**<br>Forward: 5'- ATCAGAATCCCGGTGCCGAGGCCGC<br>Reverse: 5'-TCTCAGATATCCCGTCTCGCGTATATCTGA<br>CACGTGCCTG<br>**Oligo nucleotide**<br>Forward: 5'- TA**M**GCATCGATGCAGG<br>Reverse: 5'-CCTGCATCGATG

Name	Sequence (sense strand) and primers
5mC at -58 position on Watson strand of Widom601<br><br>**(Nuc-58W)**	ATCAGAATCCCGGT**M**GCGAGGCCGCTCAATTGGTCGT<br>AGACAGCTCTAGCACCGCTTAAACGCACGTACGCGCT<br>GTCCCCCGCGTTTTAACCGCCAAGGGGATTACTCCCTA<br>GTCTCCAGGCACGTGTCAGATATATACATCGAT (145 bp)<br><br>**Primer**<br>Forward: 5’ ATCAGAATCCCGGT**M**GCGAGGCCGC<br>Reverse: 5’- ATCGATGTATATATCTGACACGTGC
5mC at -62 position on Watson strand of Widom601<br><br>**(Nuc-62W)**	ATCAGAATCC**M**GGTGCCGAGGCCGCTCAATTGGTCGT<br>AGACAGCTCTAGCACCGCTTAAACGCACGTACGCGCT<br>GTCCCCCGCGTTTTAACCGCCAAGGGGATTACTCCCTA<br>GTCTCCAGGCACGTGTCAGATATATACATCGAT (145 bp)<br><br>**Primer**<br>Forward: 5’- ATCAGAATCC**M**GGTGCCGAGGCCGC<br>Reverse: 5’- ATCGATGTATATATCTGACACGTGC
5mC at -58 position on Crick strand of Widom601<br><br>**(Nuc-58C)**	ATCAGAATCCCGGTGCCGAGGCCGCTCAATTGGTCGT<br>AGACAGCTCTAGCACCGCTTAAACGCACGTACGCGCT<br>GTCCCCCGCGTTTTAACCGCCAAGGGGATTACTCCCTA<br>GTCTCCAGGCACGTGTCC**G***ATATATACATCGAT (145 bp) *5mC on Crick strand<br><br>**Primer**<br>Forward: 5’- ATCAGAATCCCGGTGCCGAGGCCGC<br>Reverse: 5’- ATCGATGTATATAT**M**GGACACGTGC
5mC at -64 position on Crick strand of Widom601<br><br>**(Nuc-64C)**	ATCAGAATCCCGGTGCCGAGGCCGCTCAATTGGTCGT<br>AGACAGCTCTAGCACCGCTTAAACGCACGTACGCGCT<br>GTCCCCCGCGTTTTAACCGCCAAGGGGATTACTCCCTA<br>GTCTCCAGGCACGTGTCAGATATC**G***ACATCGAT (145 bp) *5mC on Crick strand<br><br>**Primer**<br>Forward: 5’- ATCAGAATCCCGGTGCCGAGGCCGC<br>Reverse: 5’- ATCGATGT**M**GATATCTGACACGTGC

*M:5-methylcytosine, underline indicates Widom601 sequence.

Table S3. Cryo-EM data collection statics for hCDCA7:nucleosome

Data collection and processing			
	hCDCA7:Nuc-58W	hCDCA7:Nuc+75W	
	Overall	Overall	hCDCA7:linker DNA (focused map)
EMDB number	EMD-39594	EMD-38198	EMD-38199
Microscope	Krios G4 (RIKEN BDR)		
Voltage (keV)	300		
Camera	K3/BioQuantum		
Magnification	105,000		
Pixel size at detector (Å)	0.83		
Total electron exposure (e^{-}/Å^2)	51.4	60.7	
Exposure rate (e^{-}/pixel/sec)	13.7	19.0	
Exposure time (sec)	2.6	2.2	
Defocus range (μm)	0.8-1.6 (interval: 0.2)		
Number of frames	48		
Energy filter slit width	15		
Micrographs collected (no.)	19,346	4,000	
Initial particle images (no.)	8,009,770	1,652,465	672,791
Final particle images (no.)	257,342	154,998	154,998
Map resolution (Å) FSC threshold	3.00	3.18	4.83
Automation software	EPU		

Model building and refinement	
	hCDCA7:Nuc-58s
PDB number	8YV8
**Initial model**	
Nucleosome	PDB: 3LZ0
CDCA7	AF2: AF-Q9BWT1-F1 (residues 261-374)

**Model composition**	
Non-hydrogen atoms	12,248
Residues (Protein/Nucleotide/Zn)	861 / 264 / 3
**RMSDs from ideal**	
Bond lengths (Å)	0.004
Bond angles (°)	0.601
**Validation**	
All-atom clashscore	9.98
Rotamer outliers (%)	4.16
**Ramachandran plot**	
Favored (%)	97.86
Allowed (%)	2.02
Outlier (%)	0.12
